# DMT1 ubiquitination by Nedd4 protects against ferroptosis after intracerebral hemorrhage

**DOI:** 10.1111/cns.14685

**Published:** 2024-04-18

**Authors:** Bingchen Lv, Ping Fu, Miao Wang, Likun Cui, Lei Bao, Xingzhi Wang, Lu Yu, Chao Zhou, Mengxin Zhu, Fei Wang, Ye Pang, Suhua Qi, Zuohui Zhang, Guiyun Cui

**Affiliations:** ^1^ Department of Neurology The Affiliated Hospital of Xuzhou Medical University Xuzhou China; ^2^ Institute of Stroke Research, Xuzhou Medical University Xuzhou China; ^3^ School of Life Sciences, Nanjing University Nanjing China; ^4^ Department of Geriatrics The Affiliated Hospital of Xuzhou Medical University Xuzhou China; ^5^ School of Medical Technology, Xuzhou Medical University Xuzhou China

**Keywords:** DMT1, Ferroptosis, hemin, intracerebral hemorrhage, Nedd4, ubiquitination

## Abstract

**Objective:**

Neuronal precursor cells expressed developmentally down‐regulated 4 (Nedd4) are believed to play a critical role in promoting the degradation of substrate proteins and are involved in numerous biological processes. However, the role of Nedd4 in intracerebral hemorrhage (ICH) remains unknown. This study aims to investigate the regulatory role of Nedd4 in the ICH model.

**Methods:**

Male C57BL/6J mice were induced with ICH. Subsequently, the levels of glutathione peroxidase 4 (GPX4), malondialdehyde (MDA) concentration, iron content, mitochondrial morphology, as well as the expression of divalent metal transporter 1 (DMT1) and Nedd4 were assessed after ICH. Furthermore, the impact of Nedd4 overexpression was evaluated through analyses of hematoma area, ferroptosis, and neurobehavioral function. The mechanism underlying Nedd4‐mediated degradation of DMT1 was elecidated using immunoprecipitation (IP) after ICH.

**Results:**

Upon ICH, the level of DMT1 in the brain increased, but decreased when Nedd4 was overexpressed using Lentivirus, suggesting a negative correlation between Nedd4 and DMT1. Additionally, the degradation of DMT1 was inhibited after ICH. Furthermore, it was found that Nedd4 can interact with and ubiquitinate DMT1 at lysine residues 6, 69, and 277, facilitating the degradation of DMT1. Functional analysis indicated that overexpression of Nedd4 can alleviate ferroptosis and promote recovery following ICH.

**Conclusion:**

The results demonstrated that ferroptosis occurs via the Nedd4/DMT1 pathway during ICH, suggesting it potential as a valuable target to inhibit ferroptosis for the treatment of ICH.

## Graphical abstract

Upon ICH, the brain's DMT1 increased but decreased with Lentivirus‐induced Nedd4 overexpression, implying a negative correlation. Nedd4 interacted with DMT1 at lysine residues 6, 69, and 277, resulting in ubiquitination and degradation of DMT1. Nedd4 overexpression relieved ferroptosis and aided ICH recovery, as indicated by functional analysis.

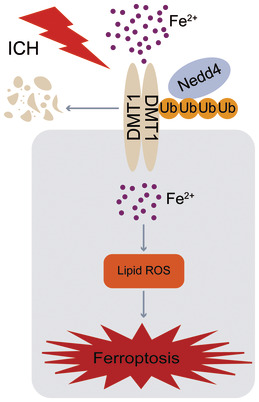

## 1 INTRODUCTION

Intracerebral hemorrhage (ICH) accounts for 10% to 15% of all strokes and carries a high morbidity and mortality rate.^1^ It is characterized by non‐traumatic intracranial bleeding resulting from the rupture of blood vessels in the brain tissues, leading to primary injury.^2^ The release of toxic blood components from the hematoma after ICH can induce secondary injury. Currently, many treatment strategies for secondary injury with ICH have not met expectations, leading to an unsatisfactory prognosis for patients.^3^ Secondary brain injury following ICH includes neuronal death, ROS accumulation, and DNA damage. At present, neuronal death and ROS accumulation are two critical aspects requiring of comprehensive investigation after ICH.^3^, ^4^ Hence, further research is necessary to understand how hematomas and their decomposition products cause these two events following ICH.

Ferroptosis, a novel form of cell death, is characterized by iron‐dependent accumulation of lipid peroxide, leading to cell death.^5^, ^6^ It has been implicated in traumatic brain injury, Parkinsonism, stroke, and other degenerative diseases.^7^, ^8^, ^9^, ^10^ Recent studies have demonstrated that mice after ICH exhibit increased levels of ROS and reduced expression of glutathione peroxidase 4 (GPX4).^10^, ^11^, ^12^, ^13^ Furthermore, the administration of the ferroptosis inhibitor, ferrostatin‐1, has been shown to attenuate brain injury after ICH.^14^ However, the specific regulatory mechanism of ferroptosis in ICH remains unclear.

Iron deposition surrounding the hematoma can lead to oxidative damage and neurotoxicity after ICH.^11^, ^15^, ^16^ Excessive iron increases the production of ROS through the Fenton reaction, causing an imbalance between ROS clearance and generation that upregulates hydroxyl free radicals and ultimately leads to ferroptosis.^17^ In addition, inhibiting iron overload has recently emerged as an effective approach to reduce ferroptosis, based on the unique iron transport mechanism.^18^, ^19^ DMT1, the first transmembrane iron transporter discovered in mammals, plays a crucial role in the transportation of Fe^2+^ and serves as a vital gateway for intracellular Fe^2+^.^20^, ^21^ These findings support a close association between DMT1 and intracellular Fe^2+^,^22^, ^23^ emphasizing the essential role of regulating DMT1 in preventing ferroptosis. A study on Parkinson's disease has revealed a correlation between ferroptosis and DMT1.^24^ In a myocardial infarction mouse model, upregulation of DMT1 promotes cell ferroptosis induced by hypoxia/reoxygenation (H/R), while DMT1 knockdown effectively inhibits H/R‐induced cell ferroptosis.^25^ Currently, increased DMT1 expression has been observed in an ICH model.^26^, ^27^ Consequently, targeting DMT1 will be an efficient method for inhibiting ferroptosis after ICH.

Nedd4 is a pivotal member of the HECT domain E3 ligase family present in eukaryotes and conserved throughout its evolution. It consists of a catalytic C‐terminal HECT domain, an N‐terminal calcium/lipid‐binding domain (C2 domain), and four WW domains responsible for cellular localization and substrate recognition.^28^ Nedd4 plays a critical role in promoting the degradation of substrate proteins and is involved in numerous biological processes. Nedd4 is widely found in various tissues and cells.^29^ As a member of the E3 ligase Nedd4 family, Nedd4 has been implicated in processes such as autophagy,^30^, ^31^, ^32^ apoptosis,^33^, ^34^, ^35^, ^36^ and necroptosis.^37^, ^38^ Recently, one study demonstrated that Nedd4 can inhibit ferroptosis in melanoma by stimulating the ubiquitination of VDAC2/3.^39^ Another study has found that robustaflavone. A strikingly induced ferroptosis by reducing the expression of Nedd4, leading to lipid peroxidation and ROS production.^40^ These studies suggest that Nedd4 acts as a negative regulator of ferroptosis.

In the present study, we observed an increase in DMT1 levels following ICH. Additionally, the binding of Nedd4 and DMT1 was decreased both in vivo and in vitro after ICH. Knockdown of DMT1 and overexpression of Nedd4 respectively attenuated ICH‐induced ferroptosis. Nedd4 inhibited neuronal death by promoting the degradation of DMT1. Furthermore, DMT1 with mutation in lysine residues 6, 69, and 277 showed inefficient degradation upon Nedd4 overexpression after ICH. In summary, our findings highlight the role of Nedd4 in mediating DMT1 ubiquitination, regulating ferroptosis, and potentially serving as a significant treatment target for ICH.

## 2 MATERIALS AND METHODS

### 2.1 Animals

Male C57BL/6J mice aged 8–12 weeks were housed in cages with a temperature of 23 ± 1°C, humidity of 40%–60%, and a 12‐h light–dark cycle (12 h of light and 12 h of darkness). Mice were housed 3–5 per cage with free access to food and water. All mice used in the study were purchased from the Animal Experimental Center of Xuzhou Medical University. The Ethical approval number is 20230T001.

### 2.2 Cell culture

HT22 cell line, BV2 cell line, C8‐D1A cell line, and HEK293T cell line were cultured in DMEM high glucose solution containing 10% fetal bovine serum and 1% penicillin–streptomycin. The cells were maintained in an incubator of 37°C containing 5% CO_2_ and 95% O_2_.

### 2.3 Induction of ICH mouse model and vitro ICH model

The ICH mouse model was constructed based on a previous study.^41^ Briefly, male mice were anesthetized with sodium pentobarbital (50 mg/kg). Then, using the stereotaxic instrument, Collagenase VII (0.045 U in 1 μL sterile saline, Sigma‐Aldrich) was injected into the striatum (AP 0.2, ML 2.5, DV −3.5) at a rate of 0.15 μL/min. After injection, 5 minutes of rest was allowed to prevent reflux. The sham group was injected with saline without collagenase. The total number of animals used in this study and mortality rates are listed in Table S1. For the ICH model in vitro, we stimulated the HT22 cell line with hemin (Sigma‐Aldrich, Germany) for 24 h to simulate in vitro stroke conditions according to our previous studies.^42^

### 2.4 Transfection of expression vectors and plasmids

HA‐tagged Ub, Ub K48, and Ub K63 were generously provided by Dr. Hengliang Shi from the Xuzhou Medical University. Other plasmids were purchased from Sangon Biotech (Shanghai, China): Flag‐tagged Nedd4 and myc‐tagged DMT1 vectors were constructed using wild‐type Nedd4 and DMT1 full‐length sequences. Construction of Flag‐tagged Nedd4ΔC2, Nedd4ΔWW, and Nedd4ΔHECT mutants was achieved by generating truncated mutants. Myc‐DMT1 K6R, Myc‐DMT1 K8R, Myc‐DMT1 K54R, Myc‐DMT1 K69R, Myc‐DMT1 K103R, Myc‐DMT1 K143R, Myc‐DMT1 K201R, Myc‐DMT1 K206R, Myc‐DMT1 K230R, Myc‐DMT1 K277R, Myc‐DMT1 K286R, Myc‐DMT1 K294R, Myc‐DMT1 K325R, Myc‐DMT1 K328R, Myc‐DMT1 K335R, Myc‐DMT1 K358R, and Myc‐DMT1 K405R mutant vectors were created by individually substituting the lysine residues at positions K6, K8, K54, K69, K103, K143, K201, K206, K230, K277, K286, K294, K325, K328, K335, K358, and K405 with arginine residues. Myc‐DMT1 3KR mutant was generated by simultaneous replacement of lysine residues at positions K6, K69, and K277 with arginine residues. Plasmids were transiently transfected into cells by using Polyplus (jetPRIME) reagent. Primers used for mutant plasmids are listed in Table S2 (capital letters indicate the mutation sites).

### 2.5 Mass spectrometry

Using the DMT1 antibody as bait, proteins bound to it were enriched and separated by the SDS–PAGE gel electrophoresis, followed by analysis using mass spectrometry (provided by Hangzhou Lc‐Bio Technologies).

### 2.6 AAV9, lentiviral, and administration

AAV‐hsyn‐p2a‐GFP and AAV‐hsyn‐nedd4‐p2a‐GFP were packaged into AAV9 by WZ Biosciences Inc (Jinan, China). Flag‐Nedd4 WT and DMT1 knockdown lentivirus were provided by Genechem (Shanghai, China). Primers used for DMT1 shRNA and Nedd4 shRNA are listed in Table S2. The injection sites (AP 0.1, ML 1.0, DV ‐3.0) for AAV9 and lentivirus.

### 2.7 Cerebral blood flow (CBF) measurement

The RWD Laser Speckle Imaging System (RFLSI III) was used to monitor changes in cerebral blood flow (CBF), as described previously.^43^ Experimental mice were anesthetized with 5% isoflurane, and the skull was surgically exposed and cleaned with hydrogen peroxide. The laser was placed 10 cm above the surface of the skull. CBF measurements were taken 1 h after ICH. The RWD LSCI software was used to calculate and analyze local CBF.

### 2.8 Hematoma area

Mice were euthanized under deep (5%) isoflurane anesthesia for quantification of hematoma volume. Coronal brain slices, 2 mm thick, were prepared using a vibratome. The brain slices were imaged using a high‐resolution imaging system. Hematoma size was calculated using Image J version 2.9.0 software.

### 2.9 Behavioral analysis

A series of neurobehavioral tests were conducted to evaluate brain damage after intracerebral hemorrhage. As previously described,^44^ the Rotarod test involved placing experimental mice in a rotating drum (ZH‐600B, Zhenghua Bio) that accelerated from 5 to 40 rpm within 5 min. Each mouse underwent three consecutive tests for 3 days before surgery and then again on the third day after surgery, with a 15‐minute interval between each test. In the Cylinder test, the experimental mice were placed in a transparent glass cylinder (15 × 9 cm). The number of contacts made by the left forelimb, right forelimb, or both with the wall of the cylinder was counted. Forelimb use asymmetry was calculated using the following formula: contralateral forelimb use (%) = 100 × left/(left + right). The Corner Test involved using two opaque hard plastic boards (30 × 20 × 1 cm) to create a 30° angle in a small corner. Before surgery, the mice were placed in the corner ten times a day for 3 days to familiarize them with the turning motion and then again on the third day after surgery. After entering the corner, the mice would stand up and then turn around. The frequency of left turns and right turns was recorded.

### 2.10 Immunohistochemistry

The mice were euthanized under deep anesthesia with 5% isoflurane, and the retrieved mouse brain tissue was fixed in 4% paraformaldehyde and embedded in paraffin. Brain tissue sections of 3 μm were deparaffinized in xylene and dehydrated in alcohol. Immunohistochemical experiments were performed using an immunohistochemistry kit (PV‐9000, ZSGB‐BIO) following the manufacturer's instructions. After antigen retrieval, the sections were placed in a 5% hydrogen peroxide solution to block endogenous peroxidase activity. Then, the sections were incubated overnight at 4°C with primary antibodies listed in Table S3, followed by secondary antibodies corresponding to the species of the primary antibodies. The antibody binding was detected using DAB. Finally, the cell nuclei were stained with hematoxylin, and images were acquired using a Leica microscope (DM1000, LEICA).

### 2.11 Immunofluorescence

After perfusion, the mouse brains were removed and fixed in 4% paraformaldehyde for 6 h. The brains were then sectioned into 40 μm slices using a vibrating microtome (VT1000S, LEICA). After drying, the brain slices were blocked with 10% BSA at room temperature for 1 h. Following a wash with PBS, the appropriate primary antibodies (listed in Table S3) were added to the slices, which were then stored overnight at 4°C. On the next day, the slices were incubated with secondary antibodies at room temperature for 2 h, followed by a wash with PBS. Finally, the slices were mounted with a DAPI‐containing mounting medium and observed and photographed using a fluorescence microscope.

### 2.12 Protein immunoblot analysis

Tissues were extracted from cell lines or brain tissue according to experimental requirements. After adding RIPA lysis buffer (P0013B, Beyotime Biotechnology), the tissues were sonicated and then centrifuged to obtain the supernatant. The obtained supernatant was subjected to BCA protein concentration measurement. The solution was then balanced using a 5× loading buffer, boiled, and stored at −20°C. Based on the molecular weight of the protein, appropriate concentrations of upper and lower gel were selected, and the samples were added to the sample wells. The gel was run under constant pressure for 2 h, followed by electrotransfer. Subsequently, the obtained bands were incubated in skim milk at room temperature for 2 h for blocking. After washing the bands with PBST, primary antibodies were added and incubated overnight at 4°C. On the second day, after washing the bands with PBST, the bands were incubated by the secondary antibodies at room temperature for 2 h. Finally, imaging and data analysis were performed.

### 2.13 Immunoprecipitation

The brain tissue and cells were lysed for 15 minutes using Cell lysis buffer for Western and IP (P0013, Beyotime Biotechnology), followed by centrifugation at 13,000 ***g*** for 15 min at 4°C to collect the supernatant. The total protein concentration was determined using a BCA assay kit (P0010, Beyotime Biotechnology) to obtain 1 mg of protein in total. The specified primary antibody (1 μg) was added to the protein sample, and the mixture was incubated overnight at 4°C with rotation. After that, 40 μL of Protein A/G PLUS‐Agarose (sc‐2003, Santa) was added and incubated for 8 h at 4°C. IgG from the same species as the primary antibody was used as a control. The agarose beads were then pelleted by centrifugation at 4°C and washed three times with cold PBS. Finally, the beads were eluted and heated to boiling in 40 μL of 2× SDS–PAGE Sample Loading Buffer (P0015, Beyotime Biotechnology) for immunoblot analysis.

### 2.14 Quantitative PCR

According to our previous research,^45^ total RNA was extracted from each group of cells using Trizol. After quantification, the mRNA was reverse transcribed into stable cDNA using a cDNA synthesis kit (HiScript III All‐in‐one RT SuperMix Perfect for qPCR, Vazyme). According to the instructions of the qPCR kit (ChamQ Universal SYBR qPCR Master Mix, Vazyme), reagents were added and 1 μL of cDNA was added. After running 40 cycles on a PCR instrument, the data were collected for analysis. The primers used are listed in Table S4.

### 2.15 Ubiquitination analysis

After co‐transfection of the indicated plasmids and HA‐ubiquitin for 48 h, the cells were treated with a proteasome inhibitor, MG132 (20 μM), for 8 h before protein extraction. Immunoprecipitation of cell lysates was performed using primary antibodies listed in Table S3, followed by immunoblot analysis using an anti‐HA antibody.

### 2.16 Ferrous iron, MDA, GSSG, and GSH

The Ferrous Iron Colorimetric Assay Kit (E‐BC‐K773‐M, Elabscience), MDA Assay kit (KTB1050, Abbkine), GSSG Assay Kit (KTB1610, Abbkine), and GSH Assay Kit (KTB1600, Abbkine) were used for determination following the manufacturer's instructions.

### 2.17 FerroOrange

According to the manufacturer's instructions, 1 mL of diluted FerroOrange (F374, DOJINDO) solution (1 μmol/L) was added to HT22 cells in a laser confocal dish, and the cells were incubated at 37°C with 5% CO_2_ for 30 min. Images were captured using a confocal microscope (Leica STELLARIS 5, Germany).

### 2.18 Transmission Electron Microscopy

Mouse brain tissue was fixed overnight in 2.5% glutaraldehyde. Thin sections were prepared using the Leica‐EM‐UC7 ultramicrotome. The sections were then stained with 1% uranyl acetate and 0.4% lead citrate. Images were captured using the FEI Tecnai G2 Spirit TWIN transmission electron microscope.

### 2.19 ROS and lipid ROS analysis

ROS and lipid ROS levels were analyzed using the DCFH‐DA probe (S0033S, Beyotime Biotechnology) and BODIPY‐C11 dye (HY‐D1301, MCE) with flow cytometry. Following the manufacturer's instructions, the fluorescence intensity of each sample was measured using the BD Facs Canto II flow cytometer (BD Biosciences).

### 2.20 Statistical analysis

All the statistical analyses were conducted utilizing GraphPad Prism 9.5.0 (GraphPad Software, USA) and SPSS 26.0 (IBM Corporation, USA). Descriptive statistics were employed, presenting data as mean ± SD. The assumption of normality in data distribution was verified through the Shapiro–Wilk test. Two‐group comparisons involved the Student's *t* test for normally distributed continuous variables and the Mann–Whitney *U* test for non‐normally distributed variables. Multiple‐group comparisons were executed using one‐way analysis of variance (anova). Significance was determined at a two‐sided *p* < 0.05, indicating statistical significance.

## 3 RESULTS

### 3.1 DMT1 exacerbates ferroptosis after ICH

**FIGURE 1:**
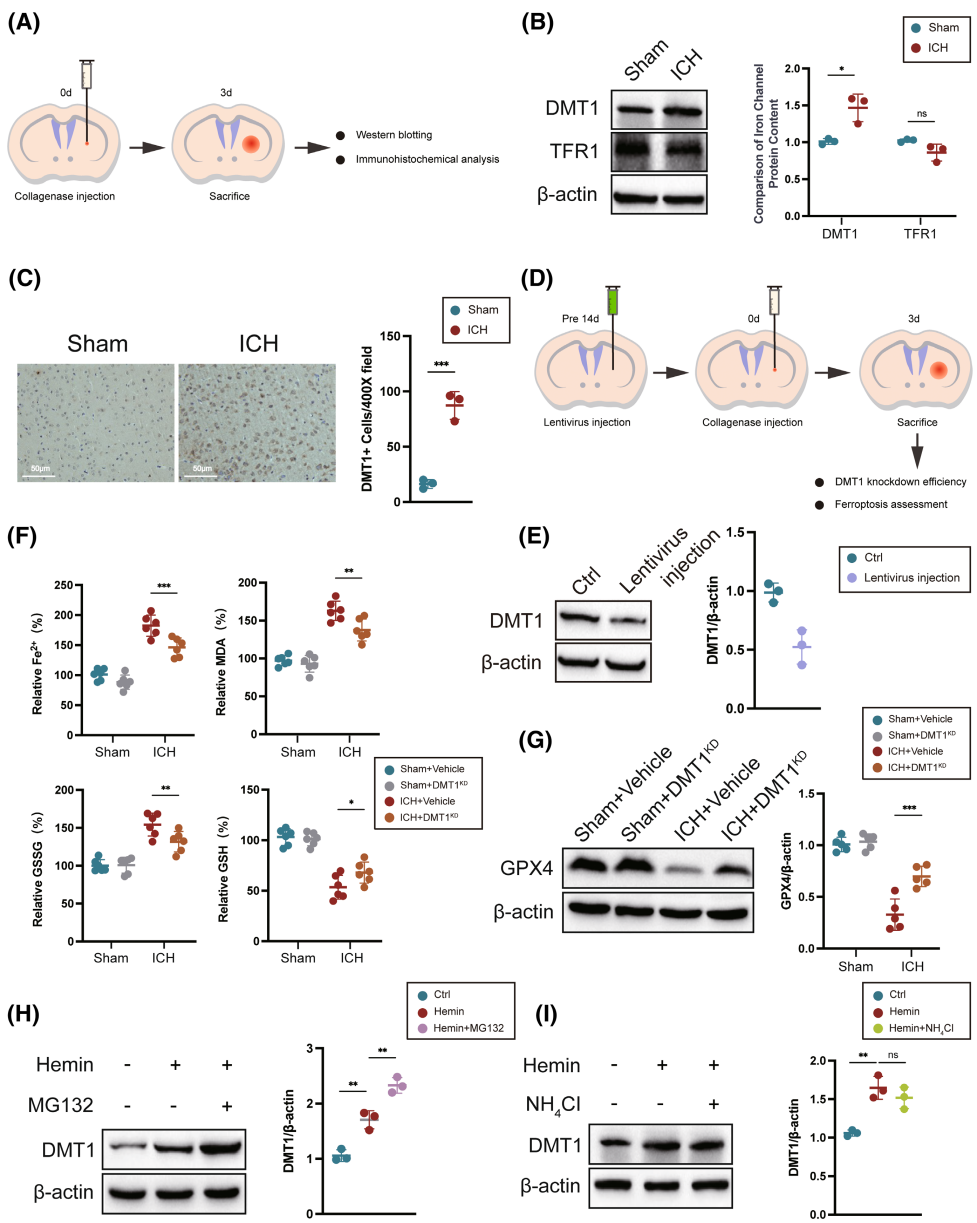
DMT1 exacerbates ferroptosis after ICH. (A) An experimental design for results is presented in (B, C). (B, C) The protein expression level of DMT1 and TFR1, and the number of DMT1‐positive cells in mice 3 days after ICH (*n* = 3/group). (D) An experimental design for results is presented in (E–G). (E) Validating the knockdown effect of DMT1 lentivirus using the western blot (*n* = 3/group). (F) Detecting the expression levels of Fe^2+^, MDA, GSSG, and GSH before and after ICH using assay kits (*n* = 6/group). (G) The protein expression level of GPX4 after ICH (*n* = 6/group). (H, I) HT22 cells were treated with Hemin (40 μM) for 24 h, followed by the addition of MG132 (20 μM) or NH4Cl (50 mM) in the culture medium for an additional 8 h. Western blot analysis was performed to detect the protein level of DMT1 (*n* = 3/group). No significance was marked by ns. *p < 0.05, **p < 0.01, ***p < 0.001.

Given that iron was transferred into the cells by the transferrin receptor 1 (TFR1) and DMT1.^46^ In our study, we established an ICH mouse model using collagenase VII (Figure 1A) and assessed the expression level of DMT1 and TFR1 at 3 days after ICH. The expression of DMT1 was significantly increased after ICH compared with the sham group, while there was no significance in TFR1 between the two groups (Figure 1B). We also observed that the number of DMT1‐positive neurons increased in 3 days after ICH (Figure 1C). These results indicated that ICH promoted DMT1 expression.

To elucidate the role of DMT1 after ICH, we constructed a DMT1 knockdown shRNA‐expressing lentivirus and administered it to the striatum of mice for 2 weeks (Figure 1D). We observed a significant decrease in DMT1 protein levels after lentivirus injection, indicating that genetic knockdown of DMT1 lentivirus was constructed successfully (Figure 1E). Previous studies have demonstrated that decreased DMT1 could ameliorate ferroptosis.^47^, ^48^ Knockdown of DMT1 reduced the level of Fe^2+^, MDA, and GSSG when compared to the vehicle group after ICH. Conversely, GSH levels and the protein level of GPX4 were increased in the DMT1 knockdown group compared with the vehicle group (Figure 1F,G). All of these data suggested that downregulating DMT1 reduced the level of Fe^2+^ and lipid hydroperoxides after ICH.

Protein post‐translational modification is a crucial way for protein degradation. To find the degradation way of DMT1, we examined whether DMT1 is regulated at the post‐transcriptional level. Using an in vitro ICH model induced by Hemin in HT22 cells, we treated the cells with either the proteasome inhibitor MG132 or the lysosome inhibitor NH4Cl. We found that DMT1 proteins were increased when treated with MG132 but not NH4Cl (Figure 1H,I), indicating that DMT1 degraded through the proteasomal pathway.

### 3.2 Nedd4 ubiquitinates and degrades DMT1

**FIGURE 2:**
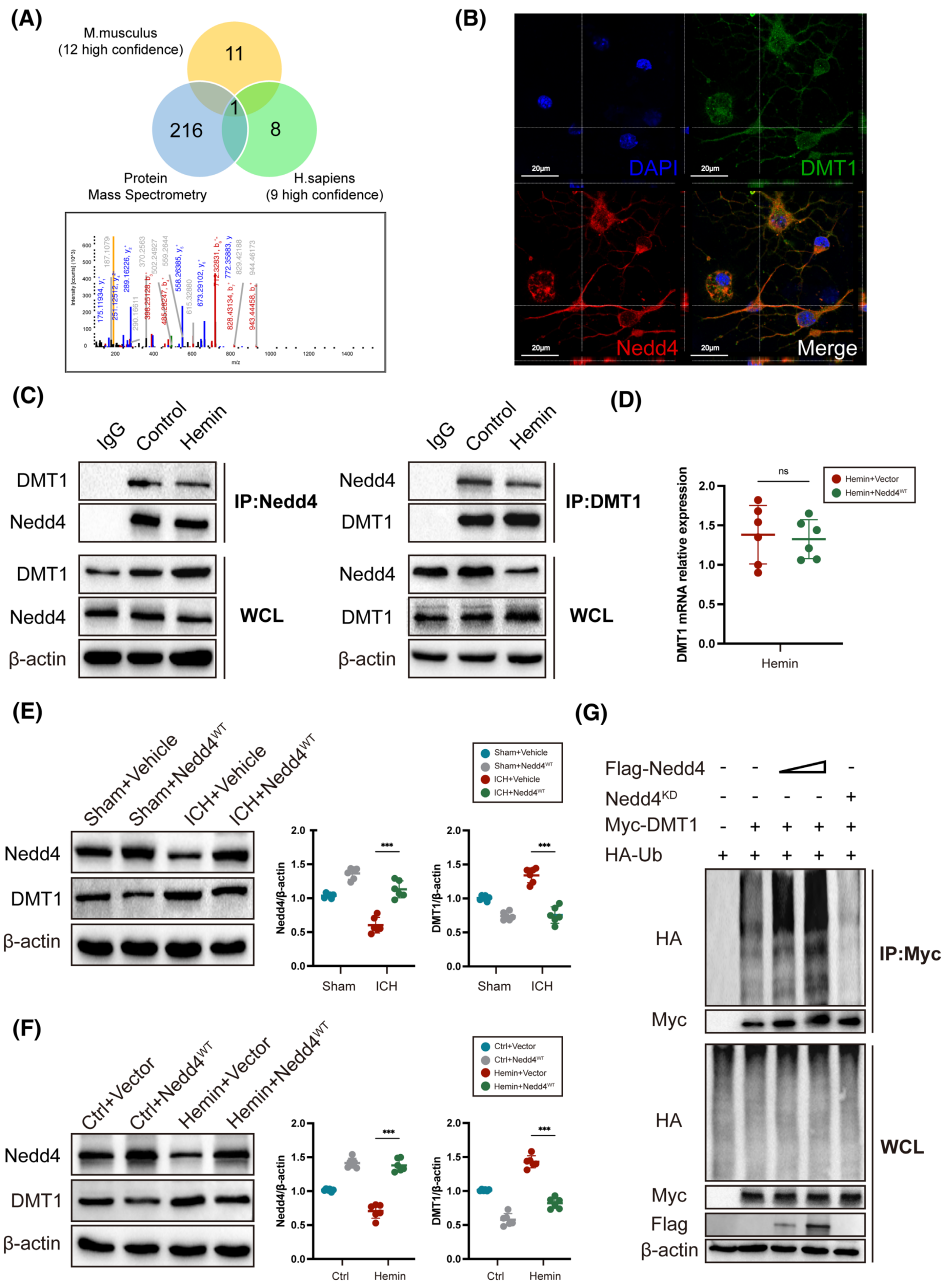
Nedd4 ubiquitinates and degrades DMT1. (A) Perform mass spectrometry analysis on the immunoprecipitation of DMT1 and intersect the results with the UbiBrowser database. (B) Immunofluorescence co‐localization image of DMT1 (green) and Nedd4 (red) in primary neurons. (C) Interactions between endogenous DMT1 and Nedd4 under basal conditions and Hemin treatment. HT22 cells were treated with either DMSO or Hemin (40 μM) for 24 h. Immunoprecipitation (IP) was performed using whole cell lysate (WCL) with control serum (IgG), anti‐Nedd4 antibody, or anti‐DMT1 antibody for detection. (D) Quantitative real‐time PCR (qRT‐PCR) was performed to detect the mRNA expression level of DMT1 in the HT22 cell line with Hemin‐induced overexpression of Nedd4 (*n* = 6/group). (E) The protein expression level of DMT1 after overexpression of Nedd4 in wild‐type (WT) mice (*n* = 6/group). (F) The protein expression levels of DMT1 in WCL after overexpression of Nedd4 in HT22 cell line (*n* = 6/group). (G) HT22 cells were transfected with the designated DNA constructs for 48 h. Prior to protein harvesting, 20 μM MG132 was added to the cells for 8 h. Cell lysates were collected and subjected to immunoprecipitation using an anti‐myc antibody. Immunoblot analysis was then performed using the specified antibodies. No significance was marked by ns. *p < 0.05, **p < 0.01, ***p < 0.001.

Next, we investigate the degradation mechanism of DMT1. First, using affinity purification and mass spectrometry techniques, we found proteins that interact with DMT1. Then, combined with the UbiBrowser database (http://ubibrowser.bio‐it.cn/ubibrowser_v3), we discovered that Nedd4, involved in the ubiquitination process, could interact with DMT1. (Figure 2A, Figure S1A,B). We then observed positive Nedd4 neurons in the brain (Figure S2A,B), and in the HT22 cell line (Figure S2C). Importantly, Nedd4 was found to colocalize with DMT1 in the primary neurons (Figure 2B). Performing co‐immunoprecipitation to validate the interaction between Nedd4 and DMT1, we found that the binding of Nedd4 and DMT1 decreased after ICH (Figure 2C). Notably, the protein level of DMT1 decreased while the mRNA levels of DMT1 had no change when Nedd4 overexpression after ICH (Figure 2D), suggesting that Nedd4 may directly regulate post‐translational instead of transcription.

Using Nedd4 overexpression lentivirus, we further elucidated the regulatory relationship between Nedd4 and DMT1. Our findings showed that the DMT1 protein declined in the Nedd4 overexpression group when compared to the vehicle group in vitro and in vivo after ICH (Figure 2E,F). Next, we co‐transfected the Myc‐DMT1 plasmid with Flag‐Nedd4 (Nedd4 overexpression) or Nedd4^KD^ plasmids (Nedd4 knockdown). We subsequently found that Nedd4 overexpression improved DMT ubiquitination, while Nedd4 knockdown reduced the ubiquitination of DMT1 (Figure 2G). Our findings suggested that DMT1 was ubiquitinated by Nedd4.

### 3.3 Nedd4 overexpression alleviates brain damage after ICH

**FIGURE 3:**
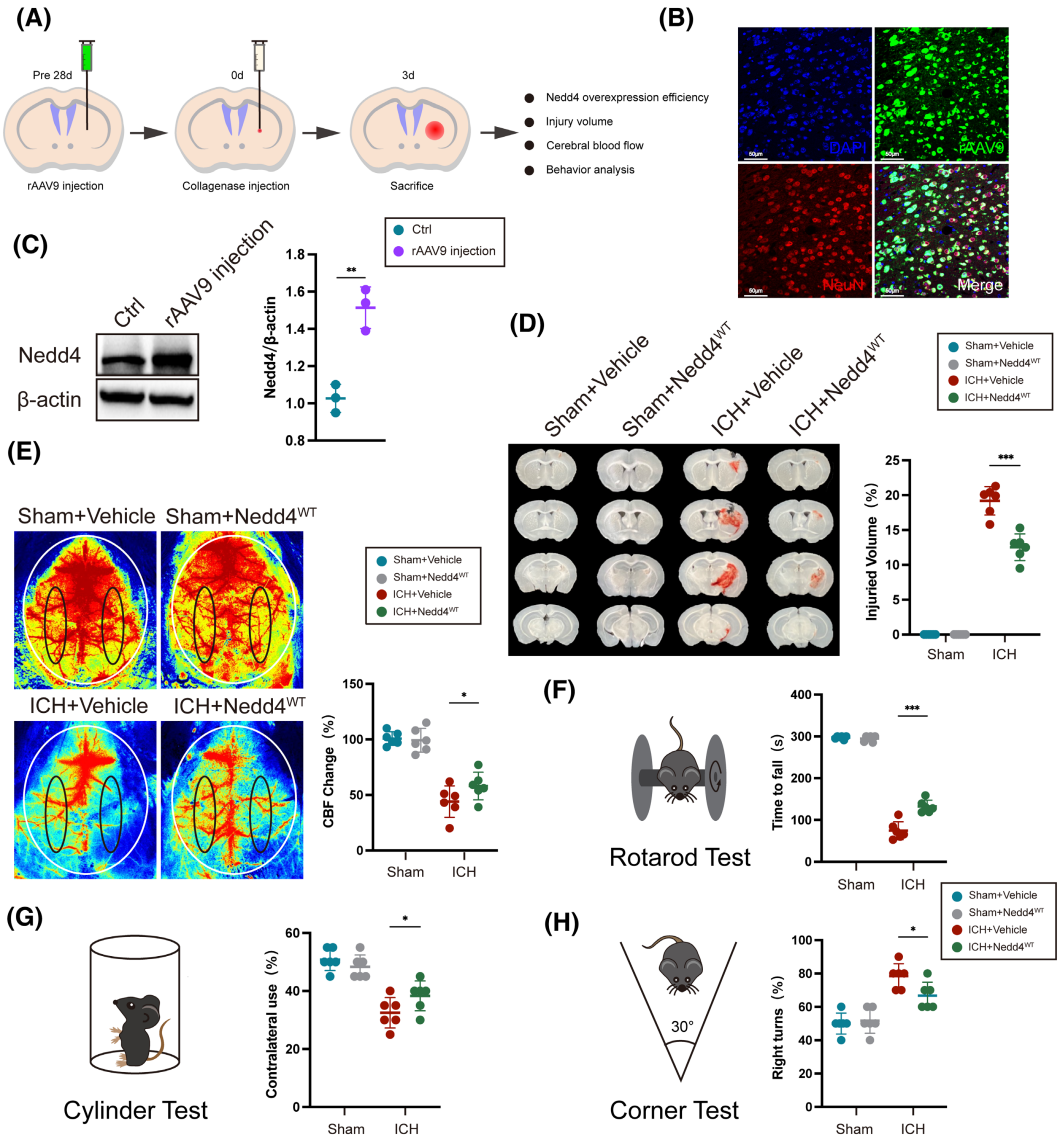
Nedd4 alleviates brain damage in mice with ICH. (A) An experimental design for results is presented in (B–H). (B) Immunofluorescence co‐localization detection of neurons (Red) and recombination adeno‐associated virus (rAAV9)‐positive cells (Green). (C) Immunoblot analysis was performed to evaluate the protein expression levels of Nedd4 in the rAAV9 injection area (*n* = 3/group). (D) Three days after ICH, the quantification of the hematoma area was performed on consecutive 2 mm coronal sections (*n* = 6/group). (E) The representative Cerebral blood flow (CBF) image was obtained 3 days after ICH. The black circle highlights the region of interest (*n* = 6/group). (F) Time to fall in the rotarod test (*n* = 6/group). (G) Contralateral forepaw use in the cylinder test (*n* = 6/group). (H) Right turns in the corner test (*n* = 6/group). *p < 0.05, **p < 0.01, ***p < 0.001.

Currently, the role of Nedd4 in ferroptosis is unclear after ICH. To discover the role of Nedd4 on neural ferroptosis after ICH. We constructed a Nedd4 overexpression mouse by injecting the rAAV9 virus of Nedd4 with the corresponding green fluorescent protein (GFP) into the brain. Following 4 weeks of injection of rAAV9, we assessed relevant markers of brain damage (Figure 3A). After a 28‐day post‐virus injection period, we verified that Nedd4 adenovirus was expressed on neurons and overexpressed Nedd4 in the protein level successfully (Figure 3B,C). Subsequently, upon induction of the ICH model, we observed that injury volume caused by ICH was significantly reduced in Nedd4 overexpression mice compared to the vehicle group (Figure 3D). Moreover, the intracerebral blood flow was augmented in the Nedd4 overexpression group compared with the vehicle group (Figure 3E). In addition, in comparison to the vehicle group with ICH, the Nedd4 overexpression group demonstrated prolonged durations in the fatigue test (Figure 3F), increased touches with the contralateral limb in the cylinder test (Figure 3G), and more turns towards the contralateral side in the corner test (Figure 3H). These results suggest that Nedd4 alleviates brain damage in mice with ICH.

### 3.4 Nedd4 overexpression protects against ferroptosis after ICH

**FIGURE 4:**
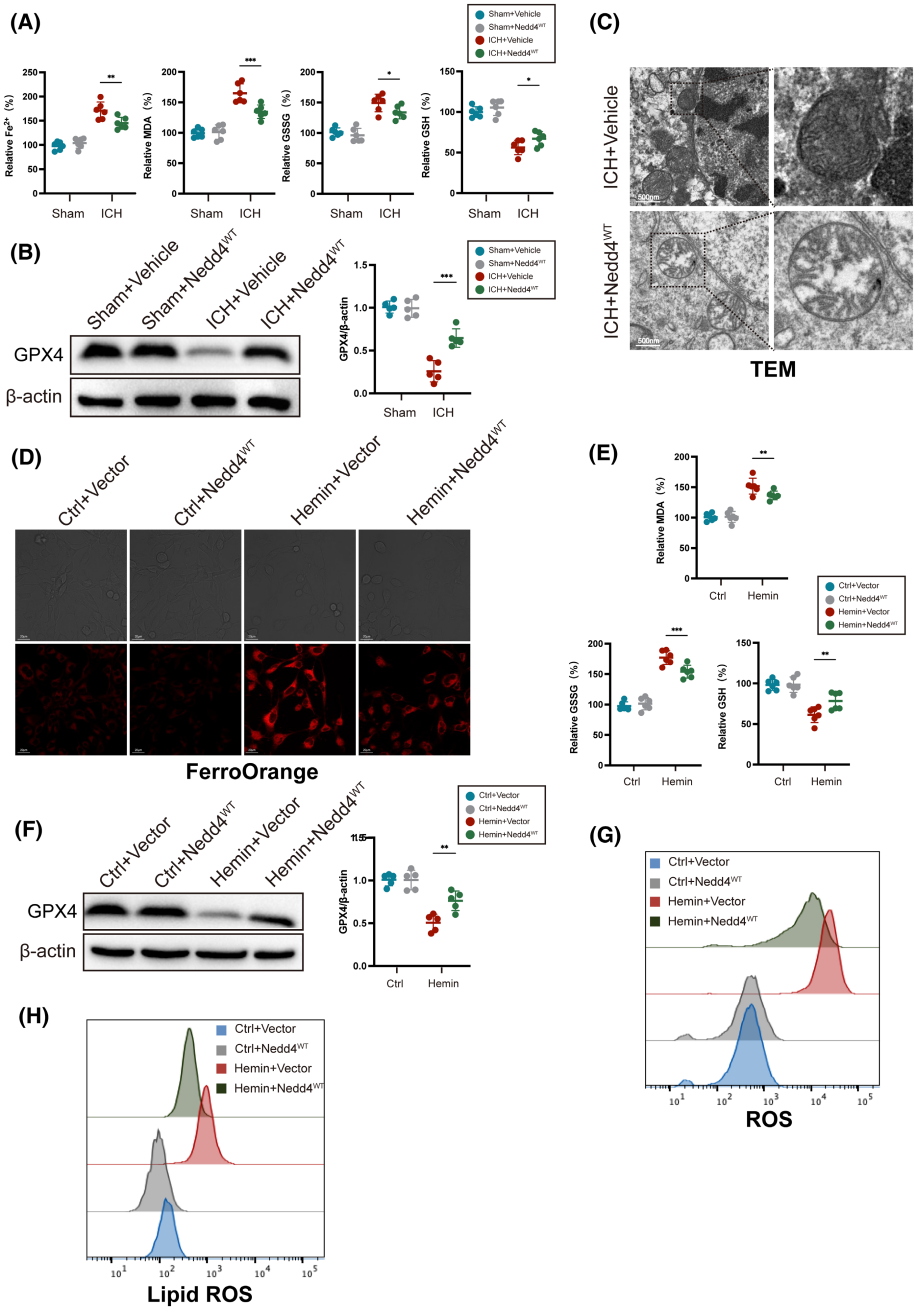
Nedd4 reduces neuronal ferroptosis after ICH. (A) The expression levels of Fe^2+^, MDA, GSSG, and GSH before and after ICH in mice injected with Nedd4 recombination adeno‐associated virus overexpression can be measured using specific assay kits (*n* = 6/group). (B) Detection of GPX4 protein expression level in mouse brain tissue using western blot (*n* = 6/group). (C) Observation of mitochondrial morphological changes using transmission electron microscopy (TEM). (D) Representative images of FerroOrange staining in HT22 cells. (E) The expression levels of Fe^2+^, MDA, GSSG, and GSH before and after Hemin treatment in the HT22 cell line can be measured using specific assay kits (*n* = 6/group). (F) Detection of GPX4 protein expression level in HT22 cell line using western blot (*n* = 6/group). (G) Flow cytometry was performed to measure ROS. (H) Flow cytometry was performed to measure lipid ROS. *p < 0.05, **p < 0.01, ***p < 0.001.

We explore the involvement of Nedd4 in ferroptosis after ICH. Administering rAAV9 carrying Nedd4 overexpression to the striatum for 4 weeks before ICH, we observed that Nedd4 overexpression decreased Fe^2+^, MDA, and GSSG levels, while GSH and GPX4 levels were increased compared to the vehicle group after ICH (Figure 4A,B). Furthermore, the Nedd4 overexpression group exhibited reduced mitochondrial membrane density, increased volume, and clearer cristae compared with the vehicle group after ICH (Figure 4C). Using a hemin‐treated HT22 cell line that overexpressed Nedd4, we found that the fluorescent intensity of iron staining was lower in the Nedd4 overexpression group compared with the vehicle group (Figure 4D). In addition, the Nedd4 overexpression group showed decreased levels of MDA and GSSG, while the levels of GSH and GPX4 increased compared to the vehicle group induced by hemin (Figure 4E,F). Moreover, the levels of ROS and lipid ROS were lower in the Nedd4 overexpression group than in the vehicle group (Figure 4G,H). These findings demonstrated the importance of Nedd4 in decreasing ferroptosis after ICH.

### 3.5 Nedd4 ubiquitinates DMT1 in a site‐specific manner

**FIGURE 5:**
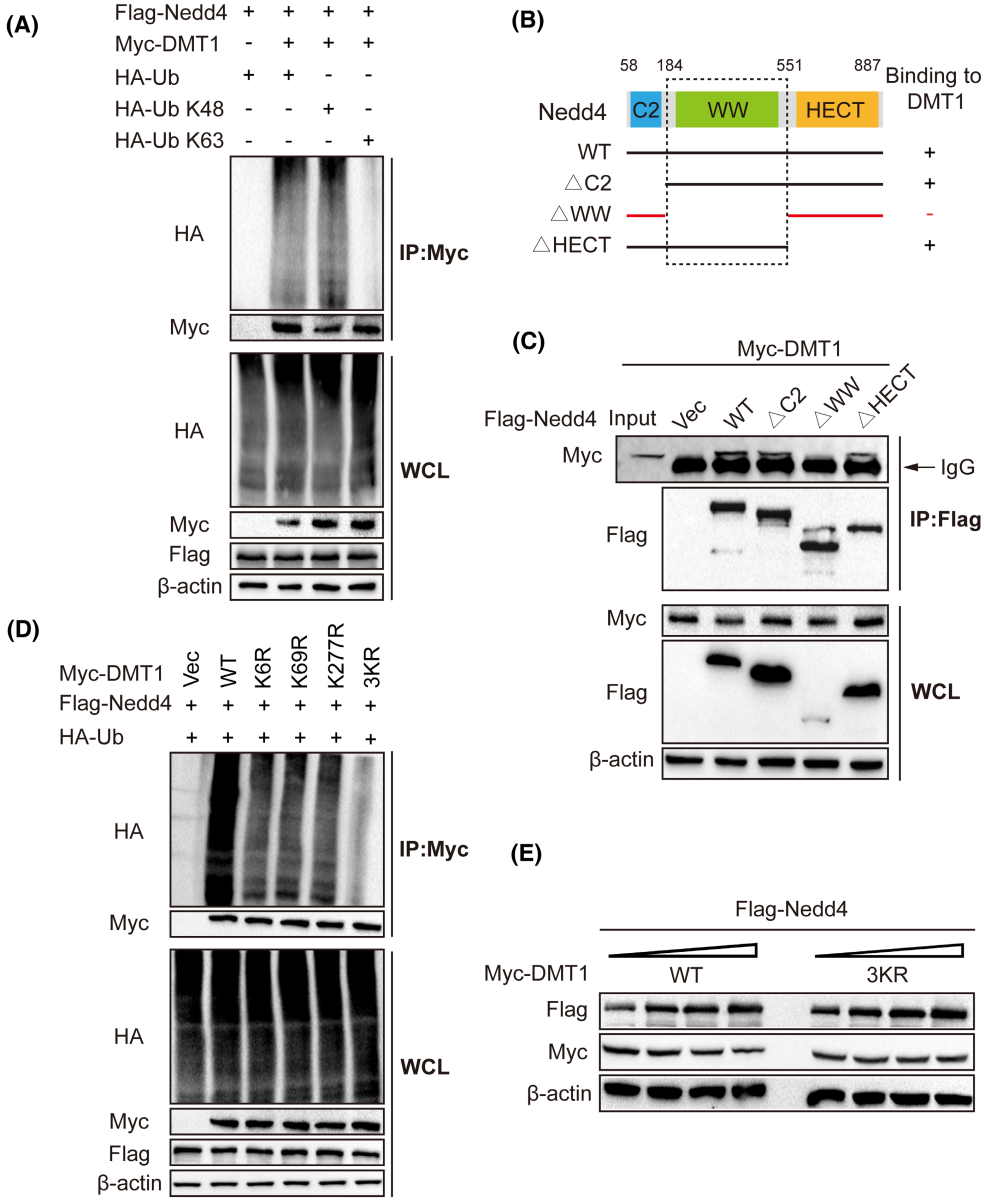
The regulatory role of Nedd4 in DMT1 degradation through specific ubiquitination sites. (A) HT22 cells were transfected with a specific DNA construct and then exposed to Hemin (40 μM) for 24 after 48 h of transfection. Subsequently, MG132 (20 μM) was added to the culture medium and incubated for an additional 8 h. The cell lysate from HT22 cells underwent anti‐myc immunoprecipitation to analyze the levels of the target protein and ubiquitination through immunoblotting. (B) A Pattern diagram to show the structure of Nedd4 and whether it mutants along with a summary of their interactions with DMT1. The plus symbol indicates binding, while the minus symbol indicates no binding. (C) Myc‐DMT1 and various Flag‐Nedd4 truncated mutants were co‐transfected into HEK293T cells. After 24 h of transfection, the cells were treated with 20 μM MG132 for 8 h. WCL were subjected to immunoprecipitation with anti‐Flag antibody, followed by immunoblotting using either anti‐Flag or anti‐Myc antibody. The WCL samples can also be used for immunoblotting analysis, with specific antibodies utilized to detect the protein expression. (D) HT22 cells were transfected with the designated DNA constructs and treated with MG132 for 8 h in one set of experiments. The lysates obtained were subjected to immunoprecipitation using an anti‐myc antibody, followed by a western blot to investigate the presence of the target protein and its level of ubiquitination. (E) HT22 cells were transfected with the designated DNA constructs and treated with Hemin (40 μM) for 24 h. The resulting cell lysates were analyzed using immunoblotting.

We transfected HEK293T cells with Flag‐Nedd4, Myc‐DMT1, HA‐Ub, HA‐Ub K48, and HA‐Ub K63 to investigate the influence of Nedd4 on ubiquitination of DMT1 induced by hemin. Treatment with hemin resulted in increased ubiquitination of Myc‐DMT1 on the K48‐linked chain, while it did no effect the ubiquitination of the K63‐linked chain (Figure 5A). Moreover, to probe the domains responsible for ubiquitination by Nedd4, we created plasmids expressing wild‐type Nedd4 with a Flag tag, as well as Nedd4 knockout constructs lacking the C2, WW, and HECT domains, respectively (Figure 5B). Co‐transfection of these mutated Nedd4 plasmids with Myc‐DMT1 plasmids into HEK293T cells, followed by immunoprecipitation using an anti‐Flag antibody, revealed that the Vec group and WW domain knockout group of Nedd4 did not interact with DMT1, whereas the WT, C2, and HECT domain knockout groups were capable of binding with DMT1 (Figure 5C). These results suggest that Nedd4 interacts with DMT1 through its WW domain.

Nedd4 was predicted as a major E3 ligase for mediating the degradation of DMT1 (Figure S1). As expected, co‐transfecting arginine substitution of individual lysine residues of DMT1 along with Nedd4 and HA‐Ub into HEK293T cells, we found that DMT1 ubiquitination decreased when mutations were substituted by the K6, K69, and K277 sites when compared to the WT group (Figure S3A). Moreover, the substitution of the three lysine residuals (K6, K69, and K277) of DMT1 was resistant to being ubiquitinated by Nedd4. (Figure 5D). Consistently, the half‐life of the DMT1^3KR^ mutant was significantly extended in HEK293T cells transfected with Flag‐Nedd4 and treated with hemin (Figure 5E), indicating that Nedd4 ubiquitinated DMT1 through the three lysine residuals. Therefore, our results identified that these three lysine residuals in DMT1 play an essential role in Nedd4‐mediated ubiquitination.

### 3.6 Nedd4 overexpression alleviates ferroptosis through the ubiquitination of DMT1

**FIGURE 6:**
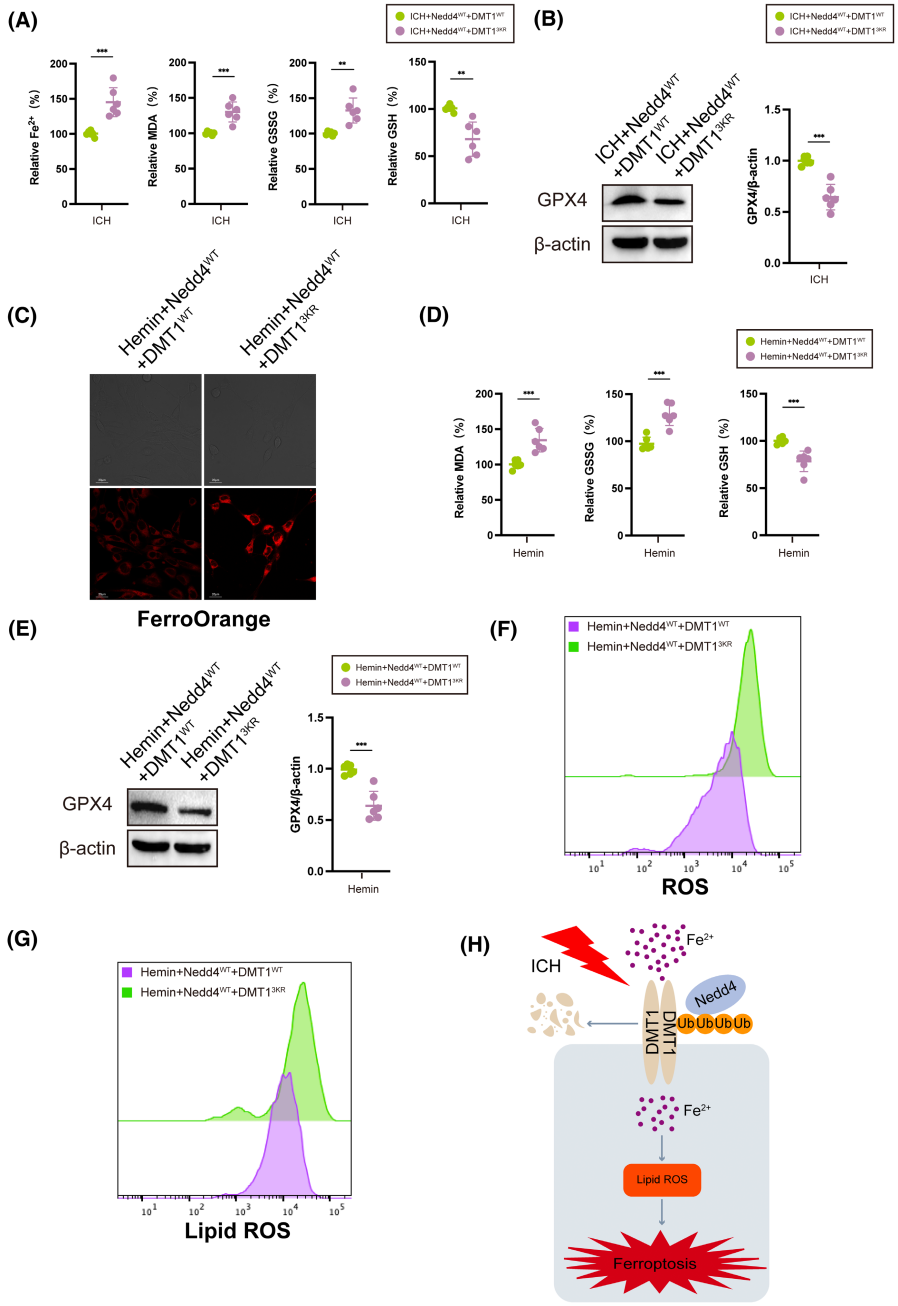
Nedd4 regulates neuronal ferroptosis mediated by DMT1 through specific ubiquitination sites. (A) The expression levels of Fe^2+^, MDA, GSSG, and GSH after ICH in mice injected with Nedd4 overexpression lentivirus, DMT1 overexpression lentivirus, and DMT1 3KR lentivirus can be measured using specific assay kits (*n* = 6/group). (B) Detection of GPX4 protein expression level in mouse brain tissue using western blot (*n* = 6/group). (C) Representative image of FerroOrange staining in HT22 cells. (D) HT22 cells were transfected with the indicated DNA constructs and incubated for 48 h. After that, the cells were treated with Hemin for 24 h. The expression levels of Fe^2+^, MDA, GSSG, and GSH in the HT22 cell line were measured using appropriate assay kits (*n* = 6/group). (E) The expression level of GPX4 (*n* = 6/group). (F) Flow cytometry was performed to measure ROS. (G) To assess the generation of lipid ROS, HT22 cells were transfected with the corresponding plasmids for 24 h and treated with 40 μM Hemin for an additional 24 h. Then, the cells were loaded with the BODIPY C11 probe for 1 h and analyzed using flow cytometry. (H) The Nedd4/DMT1 signaling pathway mediates neuronal ferroptosis induced by divalent iron after intracerebral hemorrhage. During ICH injury, the interaction between Nedd4 and DMT1 weakens, leading to a decrease in Nedd4‐mediated ubiquitination of DMT1 at the K6, K69, and K277 sites. This ultimately leads to an increase in lipid ROS, thus exacerbating neuronal ferroptosis. *p < 0.05, **p < 0.01, ***p < 0.001.

We investigated the biological function of the three lysine residuals in brain iron metabolism by injecting DMT1^WT^ and DMT1^3KR^ virus with Nedd4 after ICH. We found that the level of Fe^2+^, MDA, and GSSG in the DMT1^3KR^ group was higher than that in the DMT1^WT^ group after ICH, whereas GSH and GPX4 levels were decreased (Figure 6A,B). Subsequently, we cotransfected Nedd4^WT^ plasmids with DMT1^WT^ plasmids and DMT1^3KR^ plasmids into the Hemin‐induced HT22 cell line and found that the iron orange staining fluorescence intensity was higher in the DMT1^3KR^ group than in the DMT1^WT^ group (Figure 6C). Additionally, the DMT1^3KR^ group exhibited increased levels of MDA and GSSG compared to the DMT1^WT^ group, while the levels of GSH and GPX4 were diminished (Figure 6D,E). Furthermore, the DMT1^3KR^ group displayed higher levels of ROS and lipid ROS than the DMT1^WT^ group (Figure 6F,G). These results indicate that Nedd4 regulates hemin‐induced ferroptosis by ubiquitinating DMT1 through the K6, K69, and K277 sites, both in vivo and in vitro.

## 4 DISCUSSION

Ferroptosis, a recently recognized form of regulated cell death, contributes to neuronal death after ICH.^49^, ^50^ However, the molecular mechanism of ferroptosis after ICH is largely unknown. Here, we found that ICH stimulated DMT1 expression. Injection of the DMT1 knockdown virus reduced ferroptosis via decreasing Fe^2+^ level and reducing lipid peroxidation, markers of ferroptosis. In addition, Nedd4 can interact with and ubiquitinate DMT1 at lysine residues 6, 69, and 277. The level of DMT1 in the brain decreased when overexpression of Nedd4 after ICH, suggesting the negative correlation between Nedd4 and DMT1. Further study found that overexpression of Nedd4 can alleviate ferroptosis and promote recovery after ICH. Mechanistically, Nedd4 protects against ferroptosis through the degradation of DMT1 ubiquitination after ICH. Thus, targeting Nedd4 could be a valuable strategy to inhibit ferroptosis for the treatment of ICH.

Previously, there were pharmacological alterations and molecular characteristics of ferroptosis in neurons following ICH.^11^ Besides, ICH‐induced ferroptosis is evidenced by iron deposition, GPX4, ROS, as well as lipid peroxidation.^10^, ^50^, ^51^ Systemic administration of ferroptosis inhibitors can prevent brain injury after ICH.^10^, ^14^, ^52^ Combined with our research, these results showed that ferroptosis plays a significant role in brain injury and neuronal death after ICH.^2^, ^53^ To our knowledge, red blood cell lysis is followed by the release of large amounts of potentially neurotoxic iron into the brain parenchyma that leads to cytotoxicity, inflammation, and oxidative damage after ICH.^54^ As one of the most vital degradation products of a hematoma, iron contributes to neurotoxicity, leading to cell death and neurologic deficits after ICH.^51^, ^55^, ^56^ Numerous reports have also revealed the crucial role of iron in neuronal death after ICH.^18^, ^52^, ^57^, ^58^ Thus, how to inhibit cellular iron uptake still needs further investigation.

Numerous reports have also revealed that DMT1 plays a crucial role in iron transport.^59^ Previous studies have demonstrated that DMT1 expression increased after ICH,^26^, ^27^ which is consistent with our experimental findings. Specifically, our study showed an elevation in DMT1 levels and an associated increase in Fe^2+^ concentrations following ICH, indicating the potential significance of DMT1 in regulating Fe^2+^ level. Moreover, we observed that knocking down DMT1 inhibited neuronal ferroptosis, as evidenced by reductions in Fe^2+^, MDA, GSSG, and along with augmentation of GPX4 levels. The role of DMT1‐mediated ferroptosis has been explored in several systems, including subarachnoid hemorrhage,^48^ Parkinson's,^24^ and myocardial infarction.^25^ These findings imply that targeting the degradation of DMT1 could be an effective strategy for mitigating ferroptosis. Notably, a previous study showed that DMT1 could be degraded via proteasomal degradation,^60^ which is the same as our data. Therefore, our results provide another possible investigation of the degradation of DMT1, which may be mediated by decreased Fe^2+^‐dependent ferroptosis.

Nedd4, an E3 ubiquitin ligase, exerts neuronal protective functions through ubiquitination‐dependent mechanisms.^61^, ^62^ Several studies have found that Nedd4 protects against acetaminophen‐induced liver injury through the ubiquitination of VDAC1.^63^ Recently, it has been discovered that Nedd4 plays an important role in regulating ferroptosis.^39^, ^40^ Nedd4 was found to be involved in ferroptosis along with VDAC2 in lung cancer.^40^ The specific mechanism was identified as Nedd4‐mediated ubiquitination of VDAC2/3 in melanoma,^39^ which plays a crucial role in ferroptosis. However, whether Nedd4 can regulate ferroptosis after ICH is still unknown. Our results have verified that overexpression of Nedd4 reduces ferroptosis and brain damage in mice with ICH. To our knowledge, this is the first time that Nedd4 has been reported to regulate ferroptosis in ICH. Meanwhile, we found that overexpression of Nedd4 both in vitro and in vivo could downregulate the protein level of DMT1 after ICH. Nedd4 is an E3 ubiquitin ligase, we guess that Nedd4 may exert its resistance to hemin‐induced ferroptosis by mediating the ubiquitination of DMT1. In our data, we revealed that Nedd4 improved the ubiquitination of DMT1 after ICH. Thus, we speculate that manipulating the interaction between Nedd4 and DMT1 will contribute to the exploration of novel mechanisms of ferroptosis in the process of ICH.

Many proteins are degraded by a ubiquitination‐dependent mechanism. In our study, we investigated the mechanism of Nedd4‐mediated ubiquitination in regulating DMT1. We discovered that Nedd4 promoted the degradation of DMT1 through K48‐linked ubiquitination, rather than K63‐linked ubiquitination, indicating that DMT1 is regulated at posttranscriptional levels. Intriguingly, we also found that DMT1 interacted with the WW domain of Nedd4, suggesting that this domain exerts its ubiquitination role. By examining the amino acid sequence of DMT1, we found DMT1 has 17 lysine residues that could be involved in ubiquitination. Further study identified that the ubiquitination of K6R, K69R, and K277R mutants of DMT1 are decreased compared with wild‐type DMT1, suggesting the three sites mediated the stability of DMT1. Furthermore, these site mutants could increase ferroptosis after ICH. Overall, our results provide a possible molecular mechanism that the neuroprotective role of Nedd4 may be mediated by downregulating DMT1‐dependent ferroptosis.

A significant issue accompanied by avoiding neuron death is resistance to the second injury. Unfortunately, our research has revealed the neuroprotective role of Nedd4 in ferroptosis during ICH, but it lacks potential effects on other cell types (i.e., astrocytes and microglia). It should be noted that this study has only investigated ferroptosis without other ways of neuron death. In the future, we should further study the relation of these neuron death ways to find a better strategy to treat ICH. In addition, we only concentrate on the overexpression of Nedd4, which may lead to the ubiquitination of DMT1, ignoring a system increase in cellular ubiquitination, and the ubiquitination of DMT1 using Nedd4 overexpression animals still needs further investigation.

In conclusion, our study illustrates the role of Nedd4 in mediating the degradation of DMT1 ubiquitination at the K6, K69, and K277, thereby conferring neuronal protection. Notably, our findings provide the initial evidence of Nedd4 serving as an E3 ligase to ubiquitinate DMT1, thereby revealing the involvement of the Nedd4/DMT1 pathway in regulating neuronal ferroptosis after ICH (Figure 6H). Consequently, this study provides an understanding of how protein post‐translational modification regulates DMT1, alleviating brain injury after ICH.

## AUTHOR CONTRIBUTIONS

Guiyun Cui and Bingchen Lv: contributed to research design and manuscript writing. Bingchen Lv: executed most experiments. Ping Fu: contributed to data acquirement and analysis. Miao Wang: assisted in experiment completion. Likun Cui and Bingchen Lv: assisted with mouse experiments. Xingzhi Wang and Lu Yu: assisted with protein immunoprecipitation. Chao Zhou, Mengxin Zhu, Fei Wang, and Ye Pang: assisted with protein extraction and cell experiments. Suhua Qi and Zuohui Zhang: revised and polished the manuscript. All authors read and approved the final manuscript.

## FUNDING INFORMATION

This work was supported by grants from the National Natural Science Foundation of China (81571210, 81771282, 82171305, and 82301493), the Science and Technology Project of Xuzhou Health Commission (XWKYHT20220156), the Open Project of Key Laboratory of Colleges and Universities in Jiangsu Province (XZSYSKF2022021 and XZSYSKF2021015), the Science and Technology Planning Project of Xuzhou (KC20113 and KC23267), Cerebrovascular Disease Youth Innovation Fund (Z‐2016‐20‐2201) and Research and Innovation Program for Graduate Students in the Jiangsu Province (KYCX22‐2948 and KYCX23‐2985).

## CONFLICT OF INTEREST STATEMENT

Authors declare no competing financial interests.

## CONSENT FOR PUBLICATION

All authors have read and approved the manuscript.

## Supporting information


Appendix S1.

Figures S1‐S3.

Tables S1‐S4.

## Data Availability

The datasets used and/or analyzed during the present study are available from the corresponding author upon reasonable request.
