# Supplementary material for: DMT1 ubiquitination by Nedd4 protects against ferroptosis after intracerebral hemorrhage
Source: CNS Neurosci Ther. 2024 Apr 18;30(4):e14685. doi: 10.1111/cns.14685 (PMC11024684; doi:10.1111/cns.14685)
Supplement: Supplementary file 1 — Appendix S1. [file CNS-30-e14685-s001.pdf]

Full unedited blot for Figure 1B

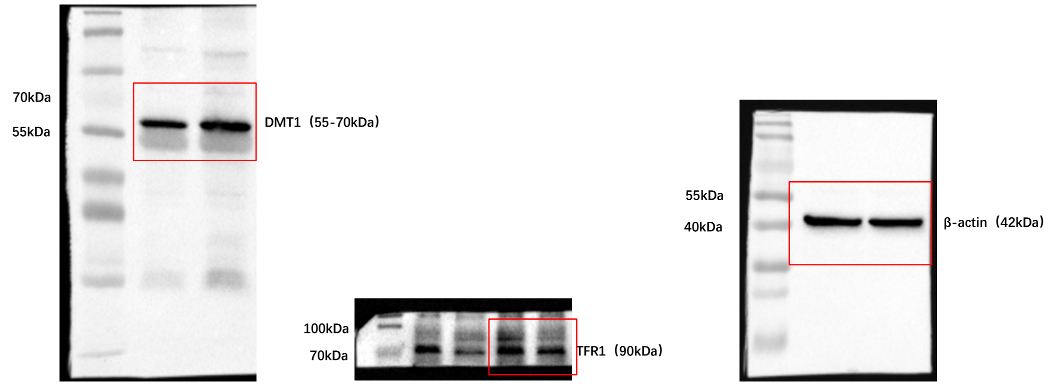

Full unedited blot for Figure 1E

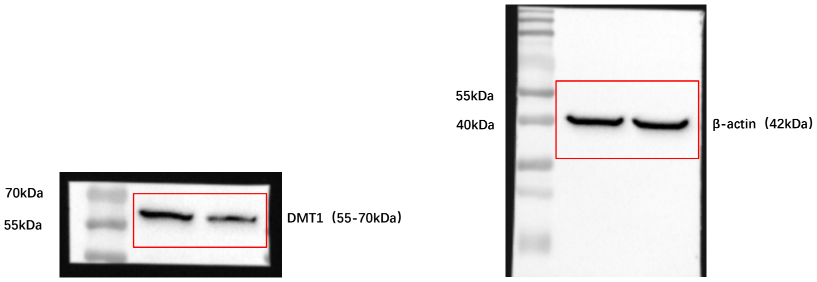

Full unedited blot for Figure 1G

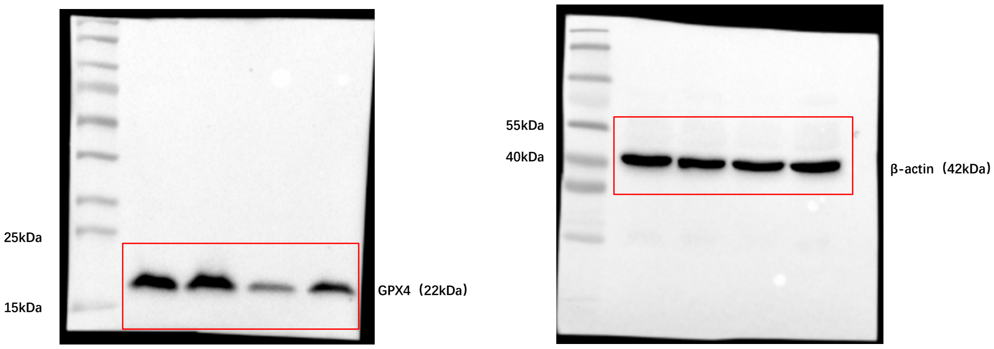

Full unedited blot for Figure 1H

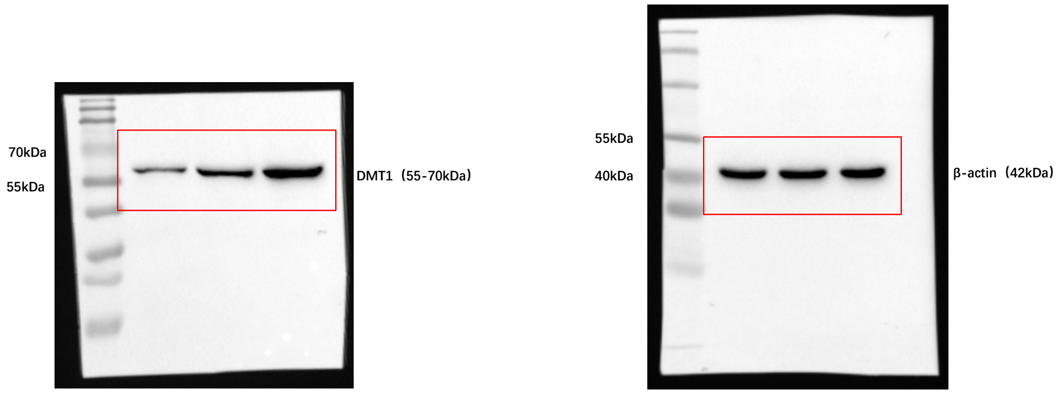

Full unedited gel/blot for Figure 1I

70kDa
55kDa

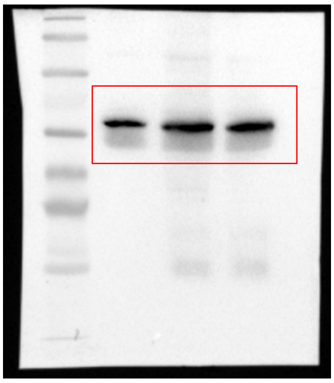

DMT1（55-70kDa）

55kDa
40kDa

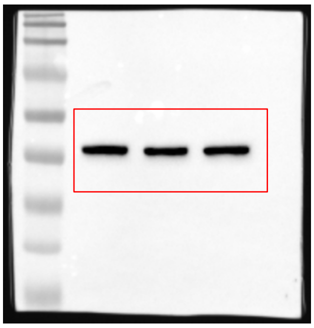

β-actin（42kDa）

Full unedited blot for Figure 2C

70kDa
55kDa

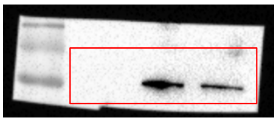

DMT1（55-70kDa）

130kDa
100kDa

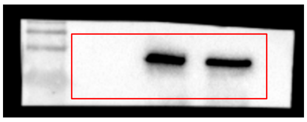

Nedd4（115kDa）

70kDa
55kDa

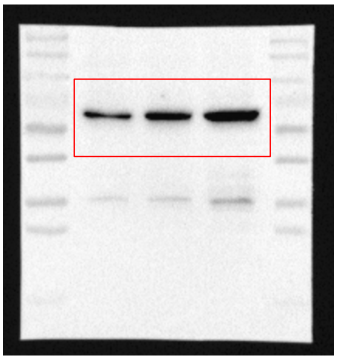

DMT1（55-70kDa）

130kDa
100kDa

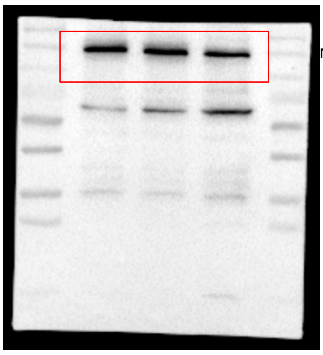

Nedd4（115kDa）

55kDa
40kDa

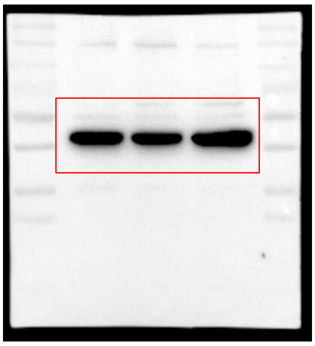

β-actin（42kDa）

130kDa
100kDa

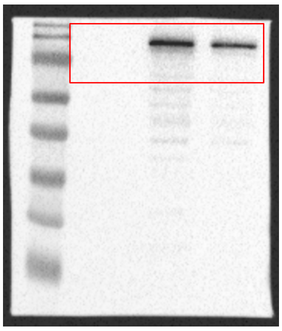

Nedd4（115kDa）

130kDa
100kDa

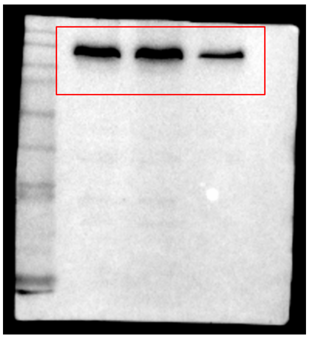

Nedd4（115kDa）

70kDa
55kDa

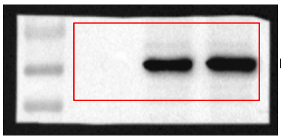

DMT1（55-70kDa）

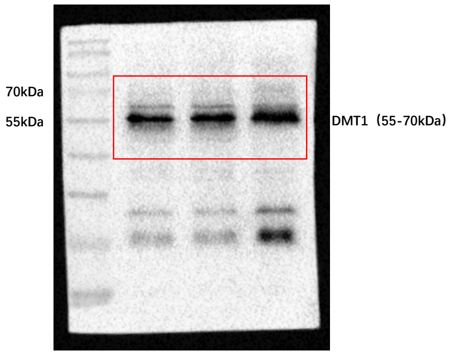

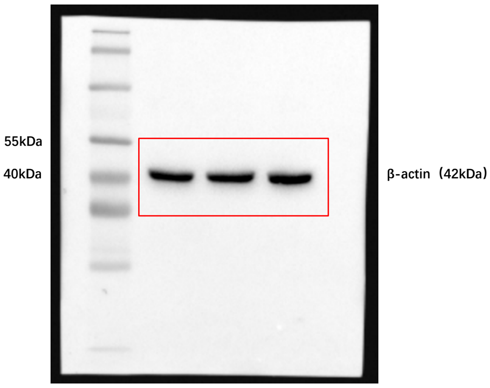

Full unedited blot for Figure 2E

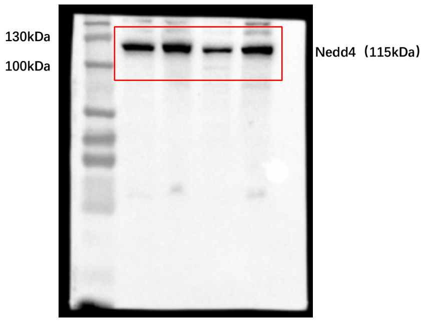

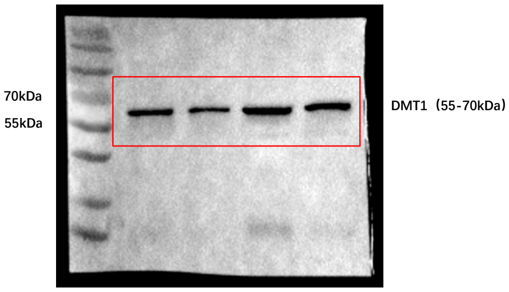

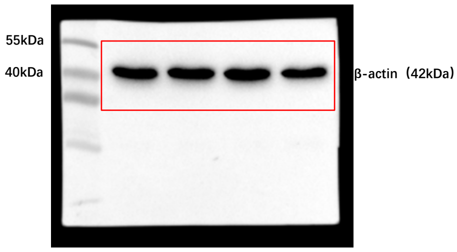

Full unedited blot for Figure 2F

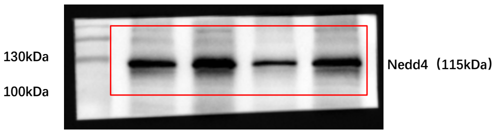

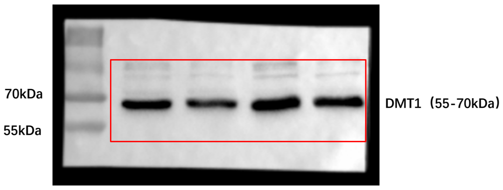

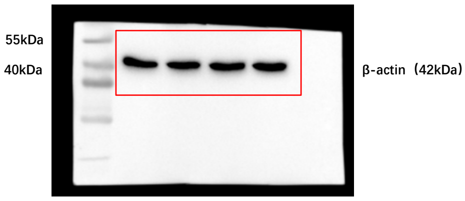

Full unedited blot for Figure 2G

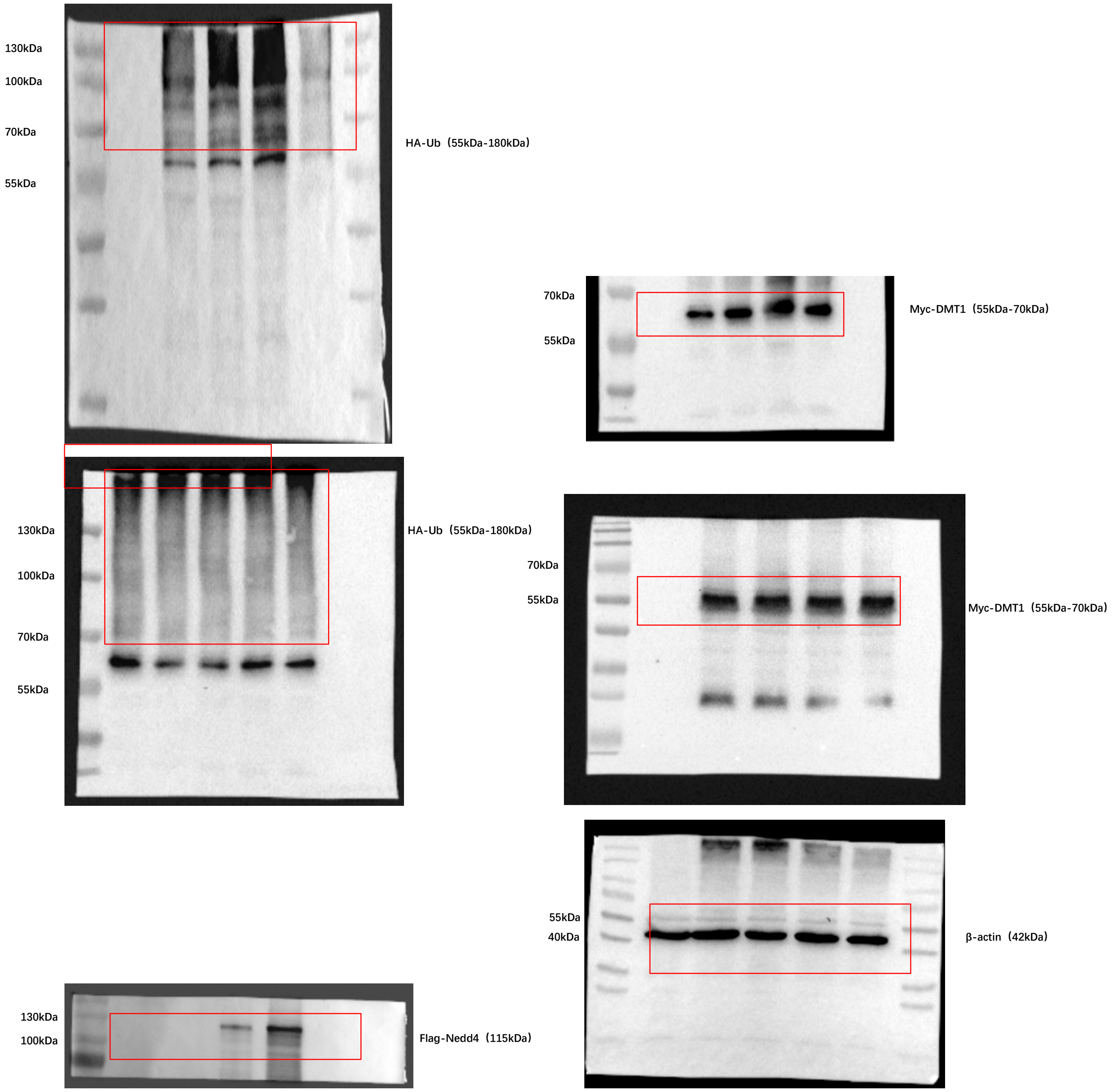

Full unedited blot for Figure 3C

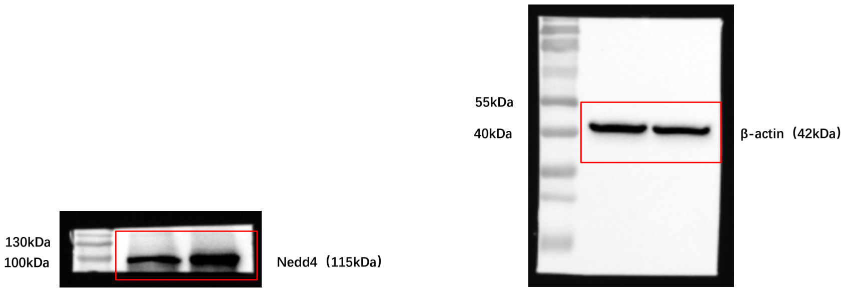

Full unedited blot for Figure 4B

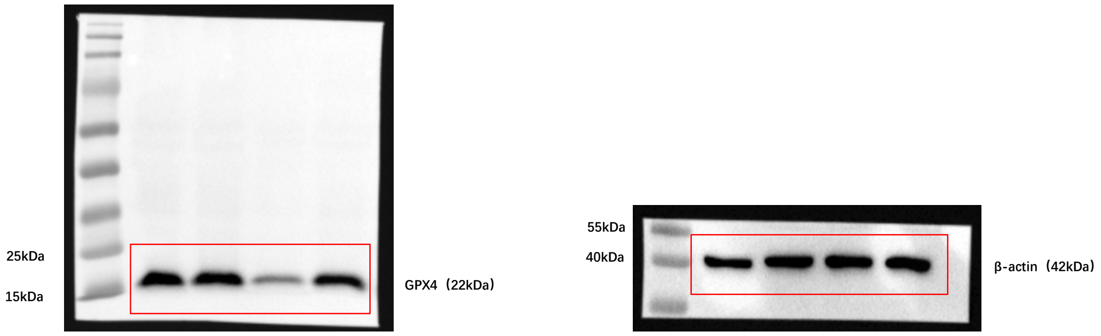

Full unedited blot for Figure 4F

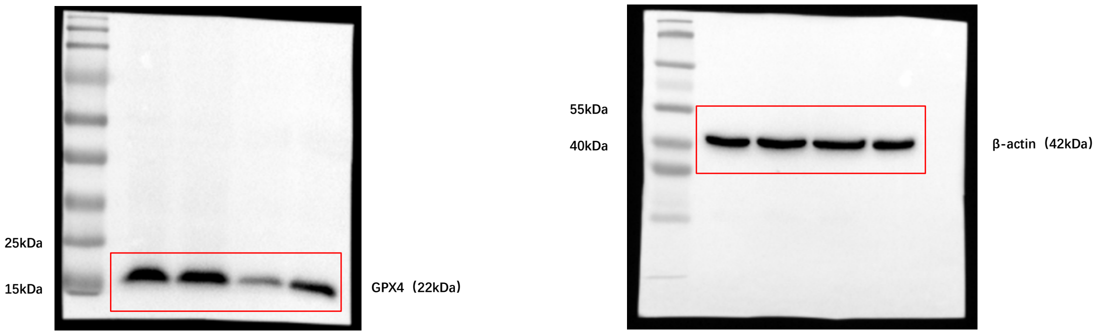

Full unedited blot for Figure 5A

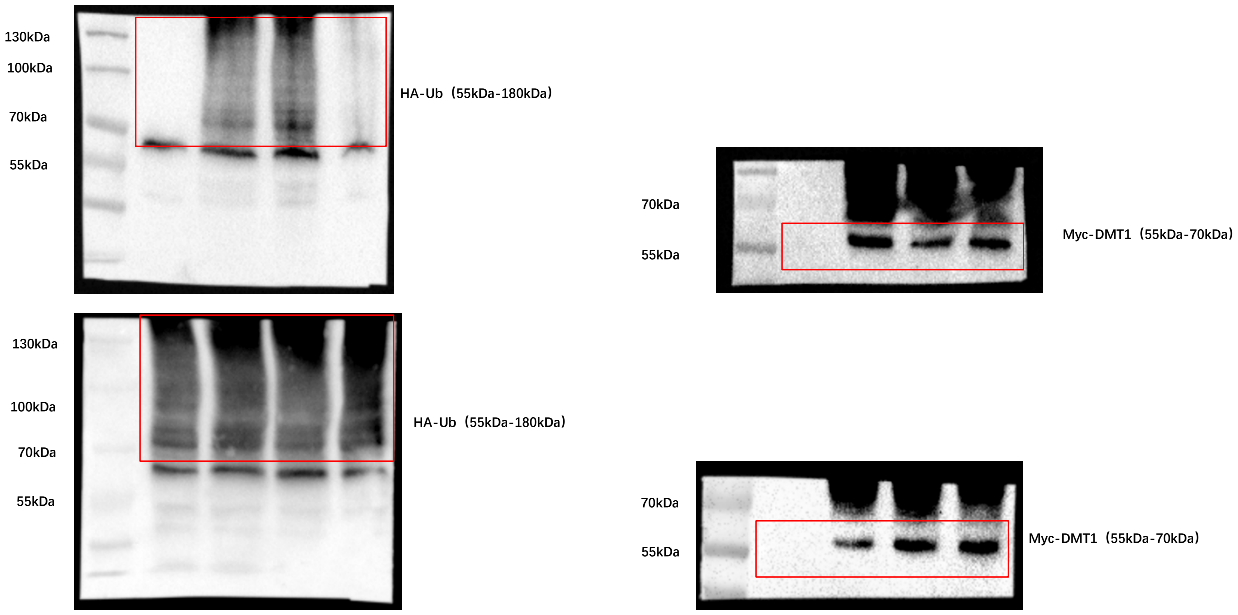

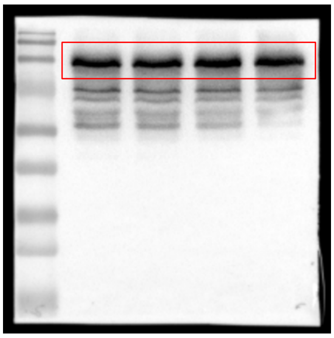

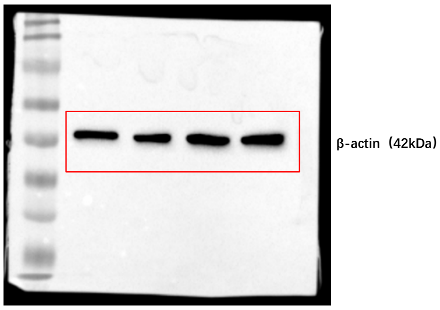

Full unedited blot for Figure 5C

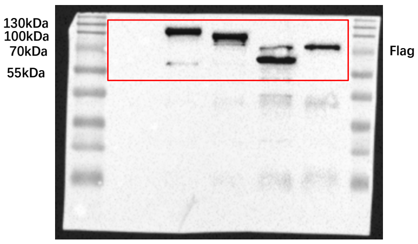

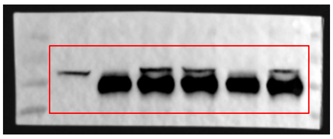

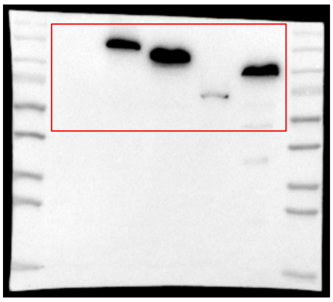

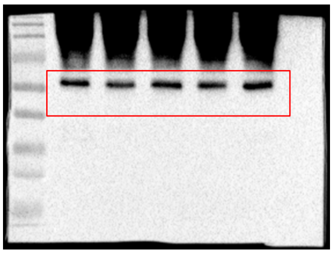

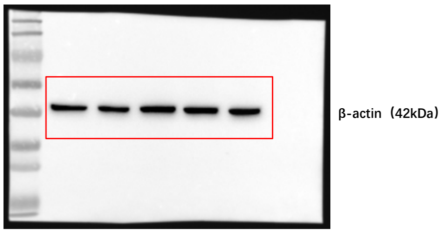

Full unedited blot for Figure 5D

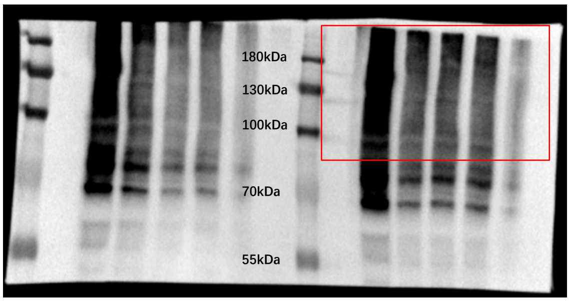

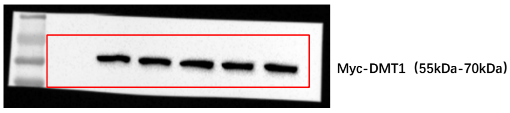

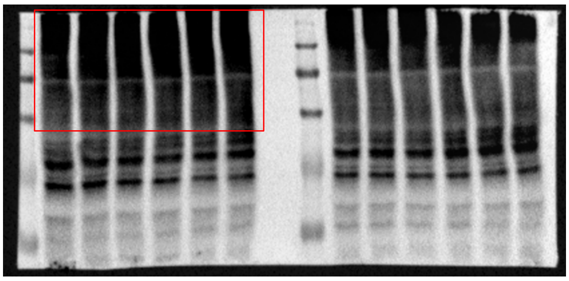

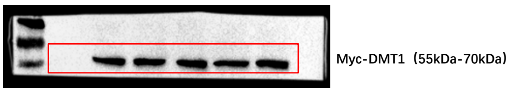

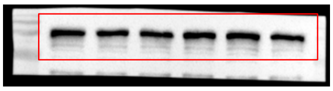

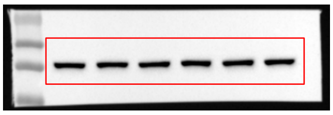

Full unedited blot for Figure 5E

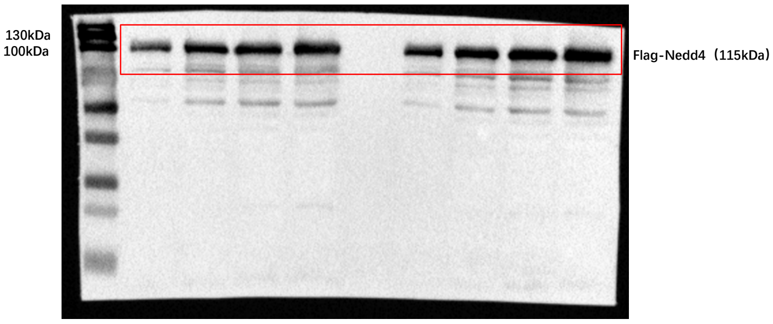

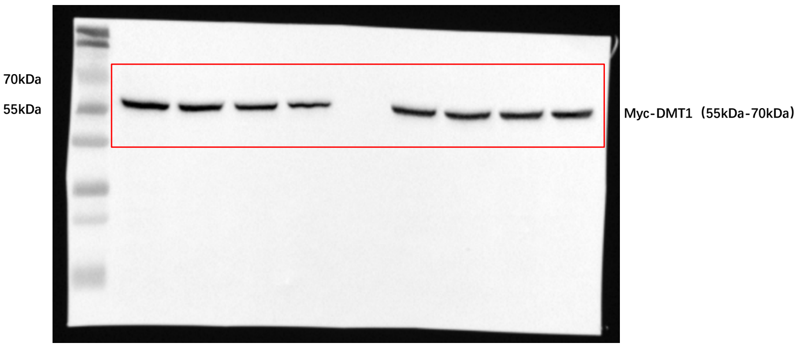

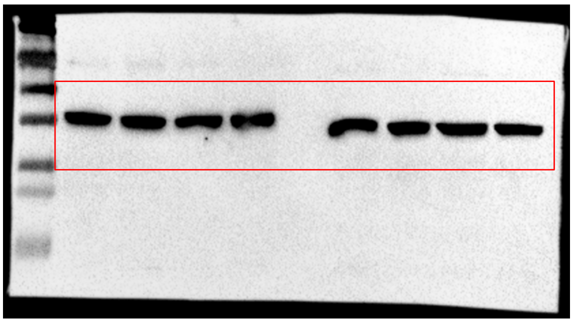

Full unedited blot for Figure 6B

25kDa
15kDa

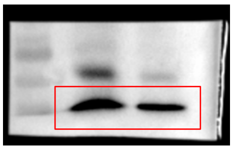

GPX4（22kDa）

55kDa
40kDa

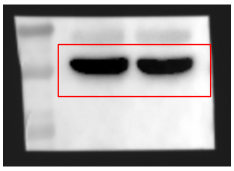

β-actin（42kDa）

Full unedited blot for Figure 6E

55kDa
40kDa

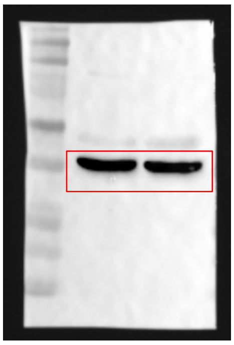

β-actin（42kDa）

25kDa
15kDa

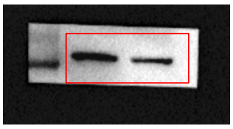

GPX4（22kDa）

Full unedited blot for Supp2B

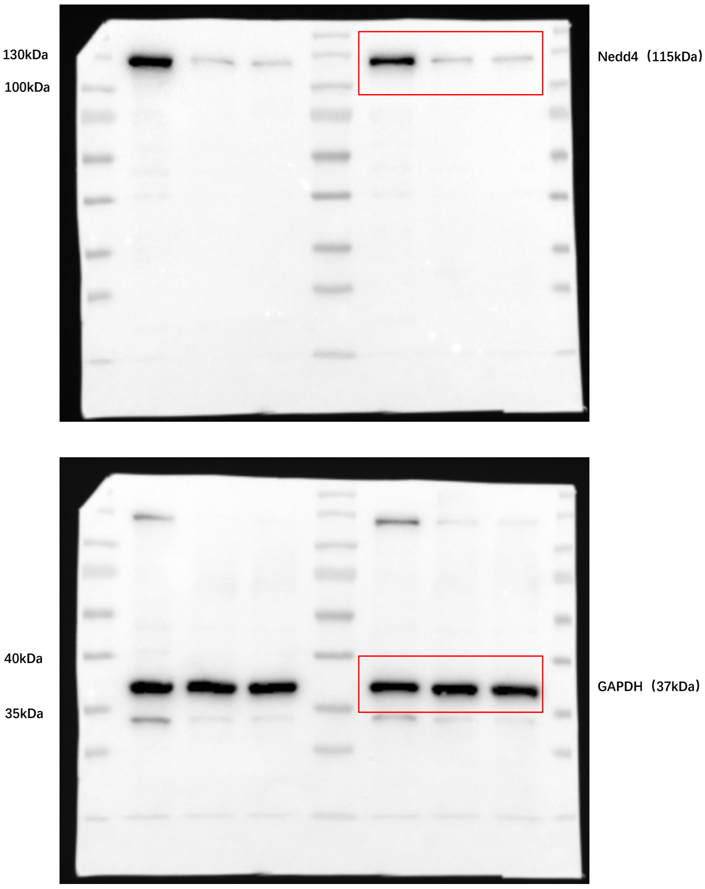

Full unedited blot for Supp2C

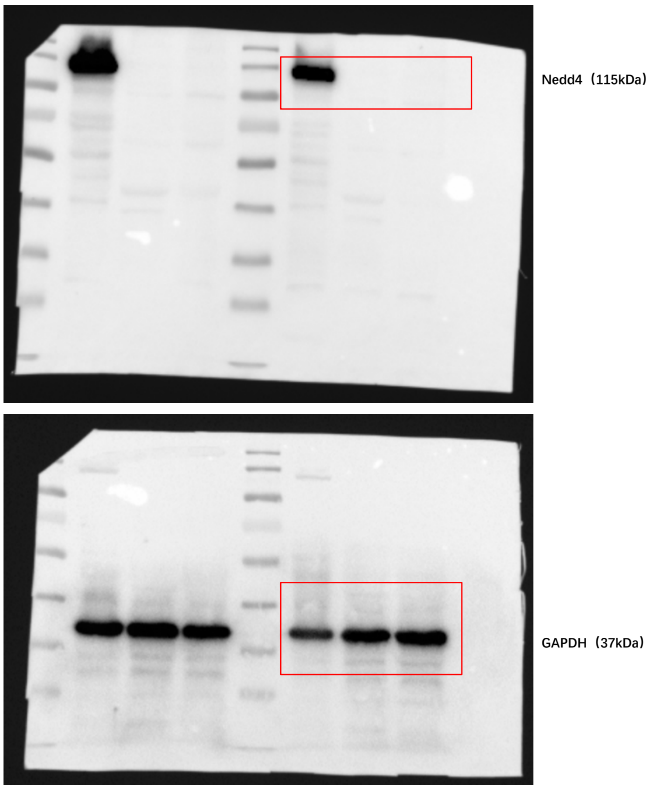

Full unedited blot for Supp3A

180kDa
130kDa
100kDa
70kDa
55kDa

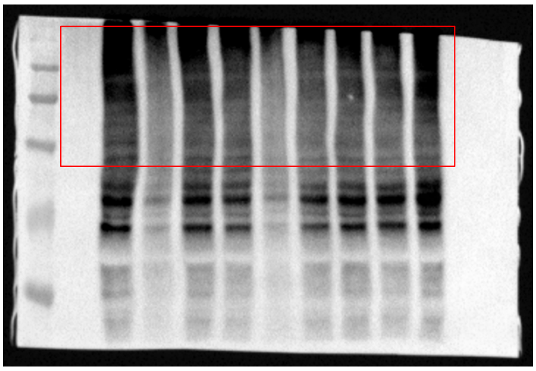

HA-Ub（55kDa-180kDa）

70kDa
55kDa

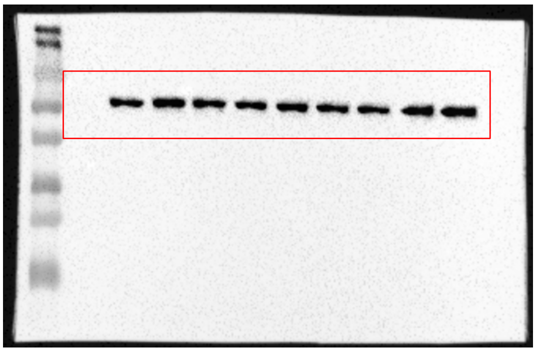

Myc-DMT1（55kDa-70kDa）

180kDa
130kDa
100kDa
70kDa
55kDa

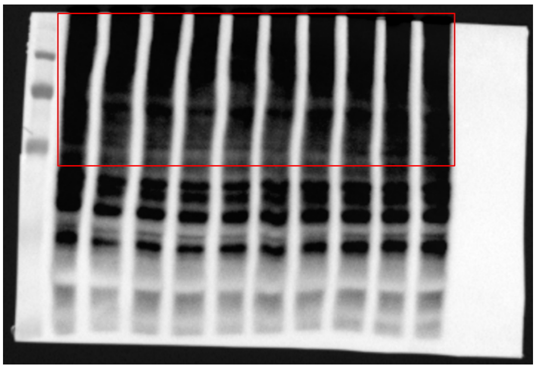

HA-Ub（55kDa-180kDa）

70kDa
55kDa

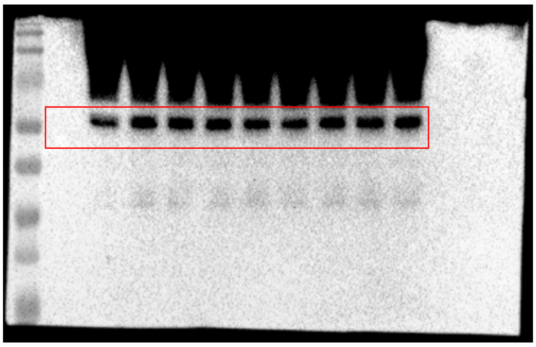

Myc-DMT1（55kDa-70kDa）

130kDa
100kDa

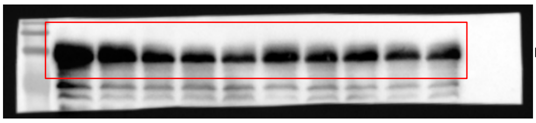

Flag-Nedd4（115kDa）

55kDa
40kDa

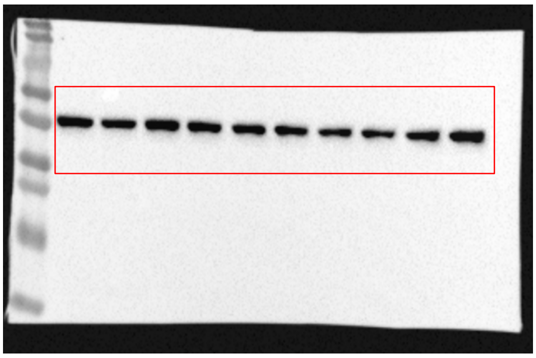

β-actin（42kDa）

180kDa
130kDa
100kDa
70kDa
55kDa

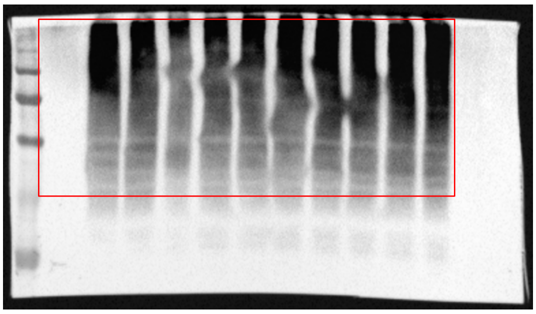

HA-Ub（55kDa-180kDa）

70kDa
55kDa

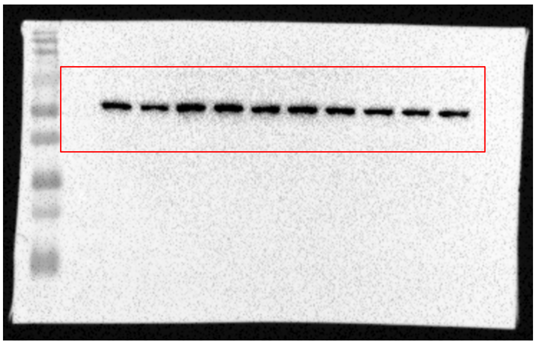

Myc-DMT1（55kDa-70kDa）

180kDa
130kDa
100kDa
70kDa
55kDa

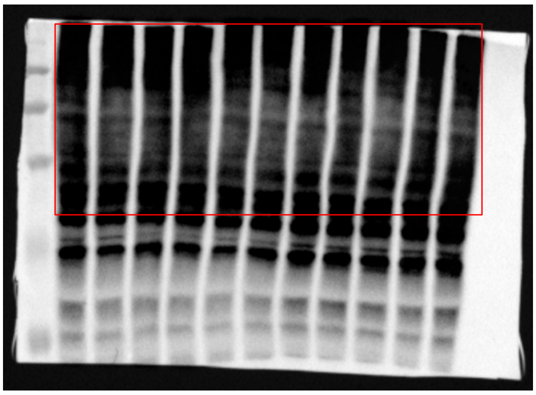

HA-Ub（55kDa-180kDa）

70kDa
55kDa

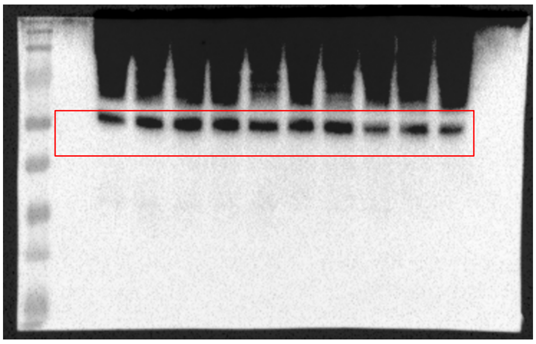

Myc-DMT1（55kDa-70kDa）

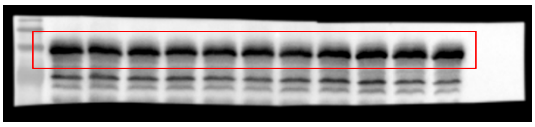
130kDa
100kDa
Flag-Nedd4（115kDa）

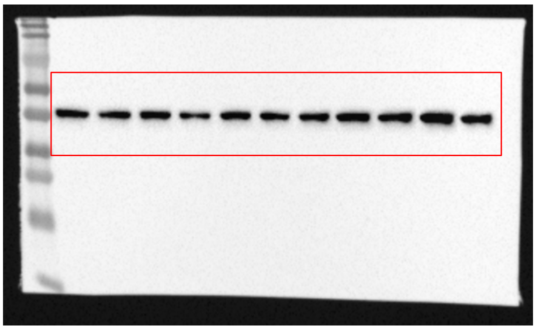
55kDa
40kDa
β-actin（42kDa）
