# Supplementary material for: DMT1 ubiquitination by Nedd4 protects against ferroptosis after intracerebral hemorrhage
Source: CNS Neurosci Ther. 2024 Apr 18;30(4):e14685. doi: 10.1111/cns.14685 (PMC11024684; doi:10.1111/cns.14685)
Supplement: Supplementary file 2 — Figures S1‐S3. [file CNS-30-e14685-s003.docx]

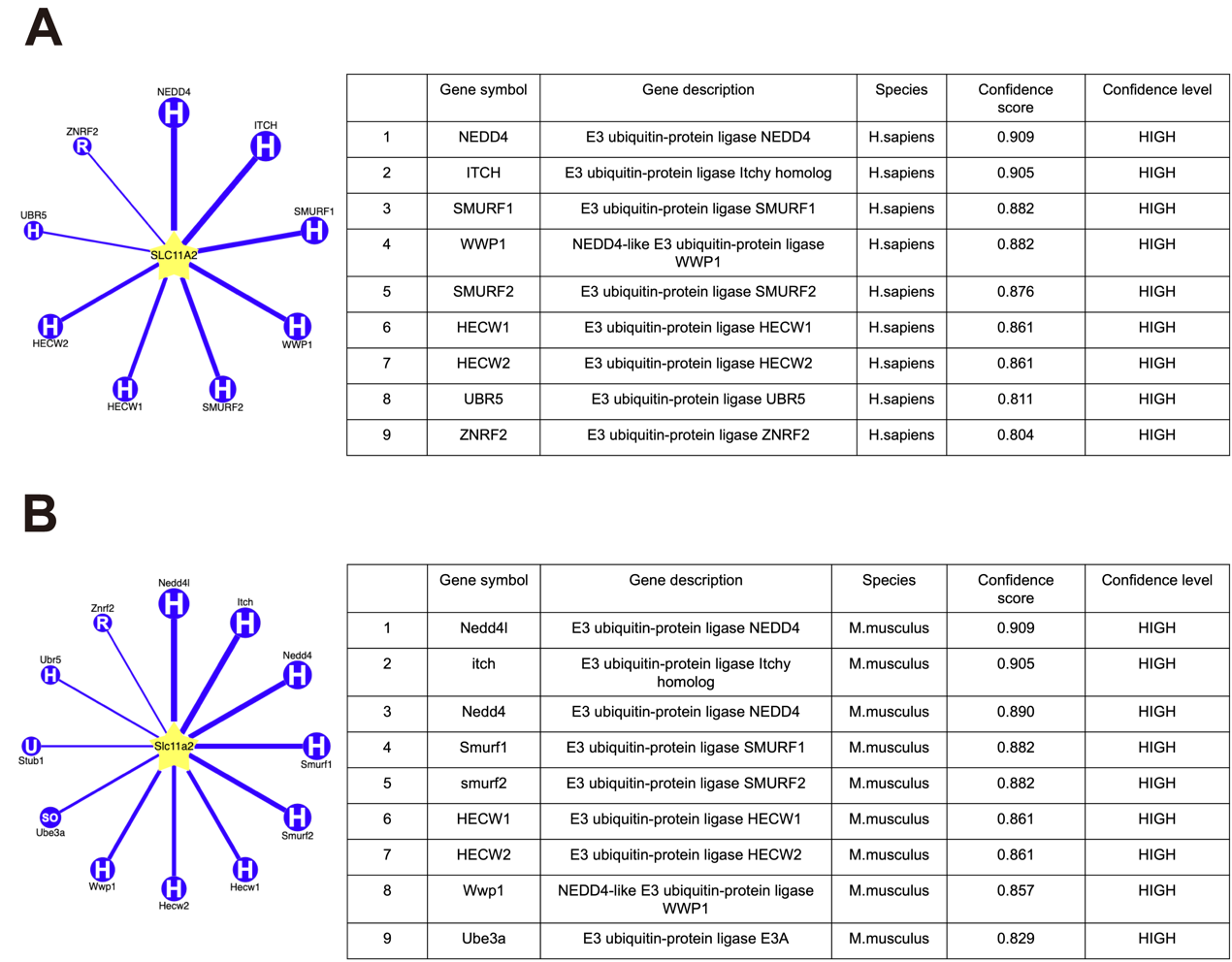

**Supplementary Figure. 1** Identification of Nedd4 as the E3 ligase of DMT1 by bioinformatics. **A-B** Nedd4 is predicted as the specific E3 ligase of DMT1 by UbiBrowser database


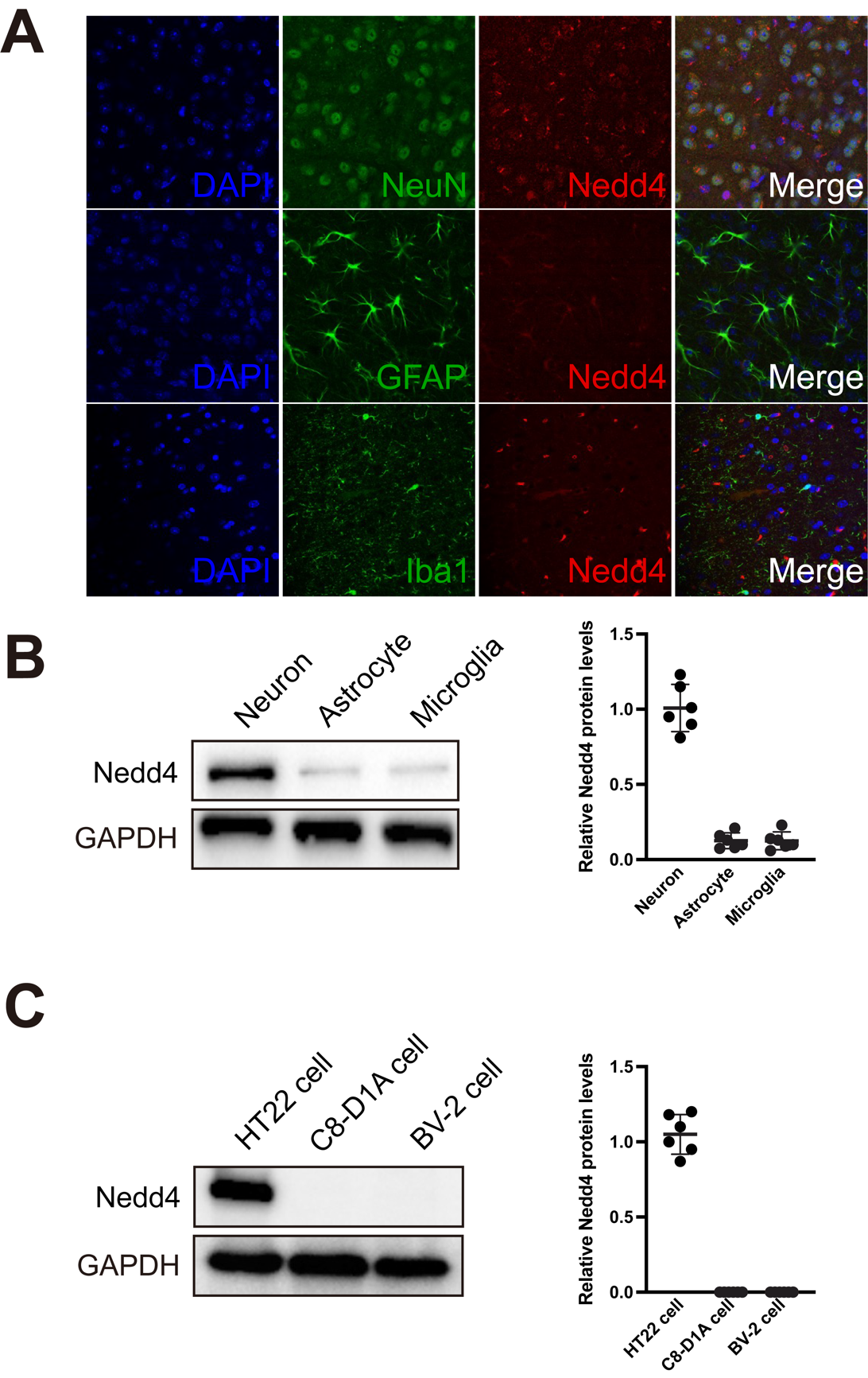

**Supplementary Figure. 2** Nedd4 is predominantly expressed in neurons of mice. **A** The co-localization of Nedd4 with NeuN, GFAP, and Iba1. **B** Western blot experiments were conducted to examine the expression profile of Nedd4 in primary neurons, primary astrocytes, and primary microglia cells derived from mice. **C** Western blot experiments were performed to detect the expression profile of Nedd4 in mouse hippocampal neuronal cell line HT22, mouse astrocyte cell line C8-D1A, and mouse microglia cell line BV-2. **P* < 0.05, ***P* < 0.01, ****P* < 0.001

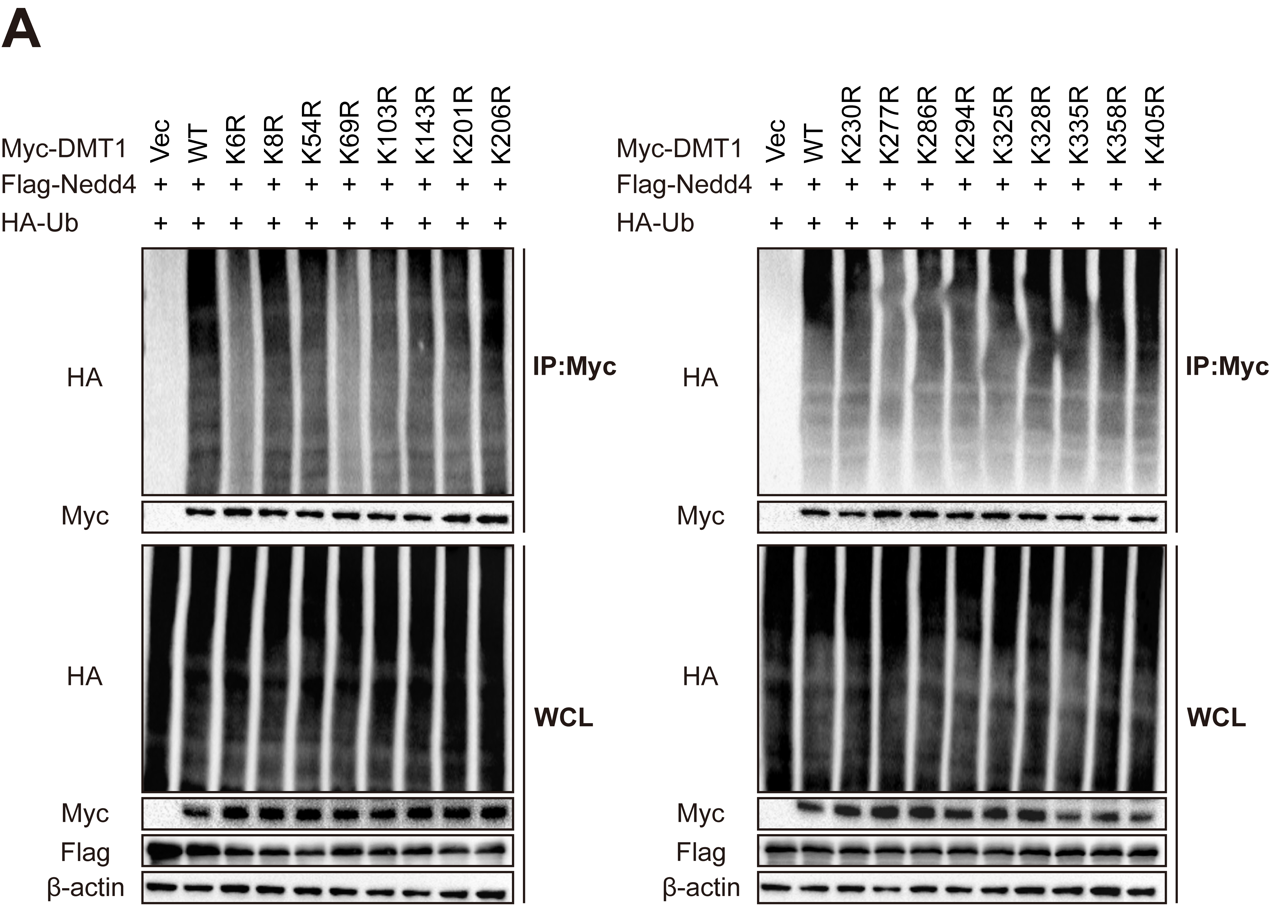

**Supplementary Figure. 3** K6、K69 and K277 of DMT1 are the critical sites for its ubiquitination. **A** HEK293T cells were transfected with DMT1 (wild-type or various KR mutants) and the HA-ubiquitin, and the cell lysates were subjected to immunoprecipitation using an anti-myc antibody, followed by Western blot analysis to detect the presence and ubiquitination levels of the indicated protein
