# Supplementary material for: DMT1 ubiquitination by Nedd4 protects against ferroptosis after intracerebral hemorrhage
Source: CNS Neurosci Ther. 2024 Apr 18;30(4):e14685. doi: 10.1111/cns.14685 (PMC11024684; doi:10.1111/cns.14685)
Supplement: Supplementary file 3 — Tables S1‐S4. [file CNS-30-e14685-s002.docx]

**Table 1**: Animal used and mortality of this study.

| **Endpoint** | **Group** | **Survival** | **Total** | **Experiment** | **Mortality (%)** |
| --- | --- | --- | --- | --- | --- |
| 3 days | Sham+Vehicle | 16 | 16 | DMT1 Knockdown efficiency  Ferroptosis assessment  GPX4 expression(WB)  NeuN,GFAP and Iba1  (IF stain) | 0.00 |
|  | Sham+DMT1^KD^ | 15 | 15 |  | 0.00 |
|  | ICH+Vehicle | 12 | 15 |  | 20.00 |
|  | ICH+ DMT1^KD^ | 12 | 13 |  | 7.69 |
| 3 days | Sham+Vehicle | 6 | 6 | Nedd4 and DMT1  expression(WB) | 0.00 |
|  | Sham+Nedd4^WT^ | 6 | 6 |  | 0.00 |
|  | ICH+Vehicle | 6 | 8 |  | 25.00 |
|  | ICH+ Nedd4^WT^ | 6 | 7 |  | 14.28 |
| 3 days | Sham | 6 | 6 | DMT1 TFR1 expression(WB)  IHC stain | 0.00 |
|  | ICH | 6 | 8 |  | 25.00 |
| Newborn mice. |  |  | 3 | Isolating primary neurons，  Primary astrocytes  and Primary Microglia |  |
| 3 days | Sham+Vehicle | 34 | 34 | Nedd4 overexpression efficiency  Injury volume  Cerebral blood flow  Behavior analysis  Ferroptosis assessment  TEM  GPX4 expression(WB) | 0.00 |
|  | Sham+Nedd4^WT^ | 33 | 33 |  | 0.00 |
|  | ICH+Vehicle | 31 | 40 |  | 22.5 |
|  | ICH+ Nedd4^WT^ | 31 | 37 |  | 16.21 |
| 3 days | ICH+Nedd4^WT^+DMT1^WT^  ICH+Nedd4^WT^+DMT1^3KR^ | 12  12 | 16  14 | Ferroptosis assessment  GPX4 expression(WB) | 25.00  14.28 |
|  | Total | 244 | 277 |  |  |

**Table 2** List of oligonucleotides used in this study

| Name | Sequence |
| --- | --- |
| Myc-DMT1^K6R^ | F 5’-atggtgttggatcctaAGAgaaaagatgcca-3’ |
| Myc-DMT1^K6R^ | R 5’-tggcatcttttctTCTaggatccaacaccat-3’ |
| Myc-DMT1^K8R^ | F 5’-ttggatcctaaagaaAGGatgccagacgatggc-3’ |
| Myc-DMT1^K8R^ | R 5’-gccatcgtctggcatCCTttctttaggatccaa-3’ |
| Myc-DMT1^K54R^ | F 5’-acctactttgatgagAGAatccccattcctgag-3’ |
| Myc-DMT1^K54R^ | R 5’-ctcaggaatggggatTCTctcatcaaagtaggt-3’ |
| Myc-DMT1^K69R^ | F 5’-tgttttagctttcgtAGActctgggcgttcacg-3’ |
| Myc-DMT1^K69R^ | R 5’-cgtgaacgcccagagTCTacgaaagctaaaaca-3’ |
| Myc-DMT1^K103R^ | F 5’-gcagtggctggatttAGGctgctctgggtgctc-3’ |
| Myc-DMT1^K103R^ | R 5’-gagcacccagagcagCCTaaatccagccactgc-3’ |
| Myc-DMT1^K143R^ | F 5’-caccgtcagtatcccAGGgtcccacggatcatc-3’ |
| Myc-DMT1^K143R^ | R 5’-gatgatccgtgggacCCTgggatactgacggtg-3’ |
| Myc-DMT1^K201R^ | F 5’-tttctttttttggacAGAtatggcttgcggaag-3’ |
| Myc-DMT1^K201R^ | R 5’-cttccgcaagccataTCTgtccaaaaaaagaaa-3’ |
| Myc-DMT1^K206R^ | F 5’-aaatatggcttgcggAGGctggaagcgtttttt-3’ |
| Myc-DMT1^K206R^ | R 5’-aaaaaacgcttccagCCTccgcaagccatattt-3’ |
| Myc-DMT1^K230R^ | F 5’-gagtacattacagtgAGGcccagccagagccaa-3’ |
| Myc-DMT1^K230R^ | R 5’-ttggctctggctgggCCTcactgtaatgtactc-3’ |
| Myc-DMT1^K277R^ | F 5’-cattctgccttagtcAGGtctagacaggtgaat-3’ |
| Myc-DMT1^K277R^ | R 5’-attcacctgtctagaCCTgactaaggcagaatg-3’ |
| Myc-DMT1^K286R^ | F 5’-gtgaatcgggccaatAGGcaggaagtgcgggaa-3’ |
| Myc-DMT1^K286R^ | R 5’-ttcccgcacttcctgCCTattggcccgattcac-3’ |
| Myc-DMT1^K294R^ | F 5’-gtgcgggaagccaatAGGtacttcttcatcgag-3’ |
| Myc-DMT1^K294R^ | R 5’-ctcgatgaagaagtaCCTattggcttcccgcac-3’ |
| Myc-DMT1^K325R^ | F 5’-gaagcattttttgagAGAaccaacaagcaggtg-3’ |
| Myc-DMT1^K325R^ | R 5’-cacctgcttgttggtTCTctcaaaaaatgcttc-3’ |
| Myc-DMT1^K328R^ | F 5’-tttgagaaaaccaacAGGcaggtggttgaagtc-3’ |
| Myc-DMT1^K328R^ | R 5’-gacttcaaccacctgCCTgttggttttctcaaa-3’ |
| Myc-DMT1^K335R^ | F 5’-tggttgaagtctgcAGAaataacagcagcccc-3’ |
| Myc-DMT1^K335R^ | R 5’-ggggctgctgttattTCTgcagacttcaacca-3’ |
| Myc-DMT1^K358R^ | F 5’-gctgtggacatctacAGAgggggtgttgtgctt-3’ |
| Myc-DMT1^K358R^ | R 5’-aagcacaacacccccTCTgtagatgtccacagc-3’ |
| Myc-DMT1^K405R^ | F 5’-ggattcctgaacctaAGAtggtcgcgctttgcc-3’ |
| Myc-DMT1^K405R^ | R 5’-ggcaaagcgcgaccaTCTtaggttcaggaatcc-3’ |
| shDMT1 Target | TACCCATCCTCACGTTCACAA |
| shNedd4 Target | ATATTCTGCTACGGATAATTA |

Primers used for the mutant DMT1 vectors and shRNAs against DMT1 and Nedd4 were listed

**Table 3** List of primary antibodies used in this study

| Antibody | Company | Lot number | Dilution ratio |
| --- | --- | --- | --- |
| Mouse anti-NRAMP2 | Santa | sc-166884 | 1:1000 |
| Rabbit anti-Nedd4 | Proteintech | 21698-1-AP | 1:2000 |
| Rabbit anti-GPX4 | Abcam | ab125066 | 1:2000 |
| Mouse anti-Beta Actin | Proteintech | 66009-1-Ig | 1:5000 |
| Mouse anti-CD71 | Santa | sc-65882 | 1:1000 |
| Mouse anti-Myc | Abcam | ab32 | 1:20000 |
| Mouse anti-Flag | Abcam | ab205606 | 1:20000 |
| Rabbit anti-HA | Proteintech | 81290-1-RR | 1:1000 |
| Mouse anti-NeuN | Cell signaling technology | #94403 | 1:500 |
| Mouse anti-GFAP | Cell signaling technology | #3670 | 1:500 |
| Mouse anti-Iba1 | Abcam | ab5076 | 1:500 |

Primary antibodies used for WB ,IF and IHC assay were listed

**Table 4** List of primers used in this study

| Gene | Primer sequences |
| --- | --- |
| DMT1 | F 5’-GTGATCCTGACCCGGTCTATCG-3’ |
| DMT1 | R 5’-TGAGGATGGGTATGAGAGCAAAGG-3’ |

The primers for Quantitative real-time PCR were listed.
